# Oleanolic acid and its analogues: promising therapeutics for kidney disease

**DOI:** 10.1186/s13020-024-00934-w

**Published:** 2024-05-30

**Authors:** Dan Pan, Yilun Qu, Chunru Shi, Cheng Xu, Jie Zhang, Hongjian Du, Xiangmei Chen

**Affiliations:** 1 The College of Traditional Chinese Medicine, Guangdong Pharmaceutical University, Guangzhou, 510006 China; 2 Department of Nephrology, First Medical Center of Chinese PLA General Hospital, State Key Laboratory of Kidney Diseases, National Clinical Research Center for Kidney Diseases, Beijing Key Laboratory of Kidney Diseases Research, Beijing, 100853 China

**Keywords:** Oleanolic acid, Kidney disease, Pharmacokinetics, Anti-inflammatory, Immunomodulatory

## Abstract

Kidney diseases pose a significant threat to human health due to their high prevalence and mortality rates. Worryingly, the clinical use of drugs for kidney diseases is associated with more side effects, so more effective and safer treatments are urgently needed. Oleanolic acid (OA) is a common pentacyclic triterpenoid that is widely available in nature and has been shown to have protective effects in kidney disease. However, comprehensive studies on its role in kidney diseases are still lacking. Therefore, this article first explores the botanical sources, pharmacokinetics, derivatives, and safety of OA, followed by a summary of the anti-inflammatory, immunomodulatory, anti-oxidative stress, autophagy-enhancing, and antifibrotic effects of OA and its analogues in renal diseases, and an analysis of the molecular mechanisms, aiming to provide further insights for the development of novel drugs for the treatment of kidney diseases.

## Introduction

Kidney diseases are classified as acute or chronic. Acute kidney injury (AKI) is characterised by a sudden decline in renal function, typically occurring within week. It is commonly observed in critically ill patients, and is associated with a high mortality rate [1]. In a large-scale retrospective study (n = 49, 147,878), the incidence of AKI in adults was found to be 21.6%, whereas that in children was 33.7%. The study also revealed that the mortality rate associated with AKI was 23.9% in adults and 13.8% in children [2]. Chronic kidney disease (CKD) is defined as a measured or estimated glomerular filtration rate (GFR) of < 60 mL/(min·1.73 m^2^) or a urine protein-to-creatinine ratio of > 30 mg/g, persisting for 90 days or longer [3]. This affects approximately 15–20% of the adult population worldwide, a significantly impacting healthcare and overall wellbeing [4]. CKD can be caused and exacerbated by various factors. Diabetes and hypertension are recognised as the leading causes of CKD [5, 6]. Additionally, research suggests that other factors such as obesity, glaucoma, second-hand smoke exposure, and HIV infection are also associated with its development [7–11]. A comprehensive assessment of CKD prevalence was conducted across 16 Asian countries, revealing an overall prevalence of 7.0–34.3%. Approximately 434 million adults in Asia are estimated to be affected by CKD, of which 65.6 million have advanced-stage CKD. Notably, the number of patients with CKD in China alone is as high as 159.8 million [12]. The global incidence of CKD increased by 88.76% from 1990 to 2016 and the number of related deaths increased by 98.02% [13]. In summary, kidney disease has gradually emerged as a global public health concern owing to its high prevalence and mortality rates, affecting over 750 million people worldwide [14].

Currently, in clinical practice, angiotensin-converting enzyme inhibitors (ACEI) or angiotensin receptor blockers (ARB) are primarily used for treating kidney disease to control blood pressure and reduce albuminuria [15]. However, ACEI and ARB have serious side effects and are contraindicated in patients with severe renal impairment [16, 17]. Thus, there is an urgent need to identify alternative therapies that are safer and more effective. In recent years, natural medicines have attracted the attention of researchers due to their multiple targets, multiple pathways, and low toxicity. Oleanolic acid (OA) is one such natural compound, numerous preclinical studies have elucidated the therapeutic effects of OA in various animal models for AKI and CKD, including renal ischemia–reperfusion injury, drug-induced renal injury, renal fibrosis, diabetic nephropathy, and lupus nephritis [18–22]. However, to date, no systematic review has evaluated the protective effects and underlying mechanisms of OA on kidney diseases. This study has attempted to address this by providing a systematic review and retrospective analysis of the basic research on the therapeutic effects of OA, its isomer ursolic acid (UA), and derivatives of OA used for treating kidney diseases. The botanical sources, pharmacological actions, pharmacokinetics, and safety aspects of OA are also discussed.

## Methodology

### Search strategy

Based on the Preferred Reporting Items for Systematic Reviews and Meta-Analyses (PRISMA) guidelines [23], this study employed a comprehensive search strategy to retrieve relevant literature from PubMed, Web of Science, Scopus, Cochrane, and Embase databases. The search keywords used were “oleanolic acid” or “ursolic acid”, which is isomer of OA frequently coexisting with it in diverse plant species owing to their structural similarity and shared pharmacological characteristics [24]. The search keywords also encompass “kidney disease”, “renal”, “nephropathy, “nephrosis”, “AKI”, “DN”, “DKD”, “CKD”, “LN”, or “IgAN”.

### Inclusion and exclusion criteria

The inclusion criteria were as follows: English research articles published between 2000 and 2023, including studies conducted on animals, cell experiments, clinical trials, and large cohort studies. The exclusion criteria were as follows: non-research articles such as reviews, meta-analyses, and letters.

### Literature screening and data extraction

Two researchers independently conducted literature searches, screening, and data extraction based on the inclusion and exclusion criteria. Any disagreements were resolved through consensus by a third researcher.

## Results

**Fig. 1:**
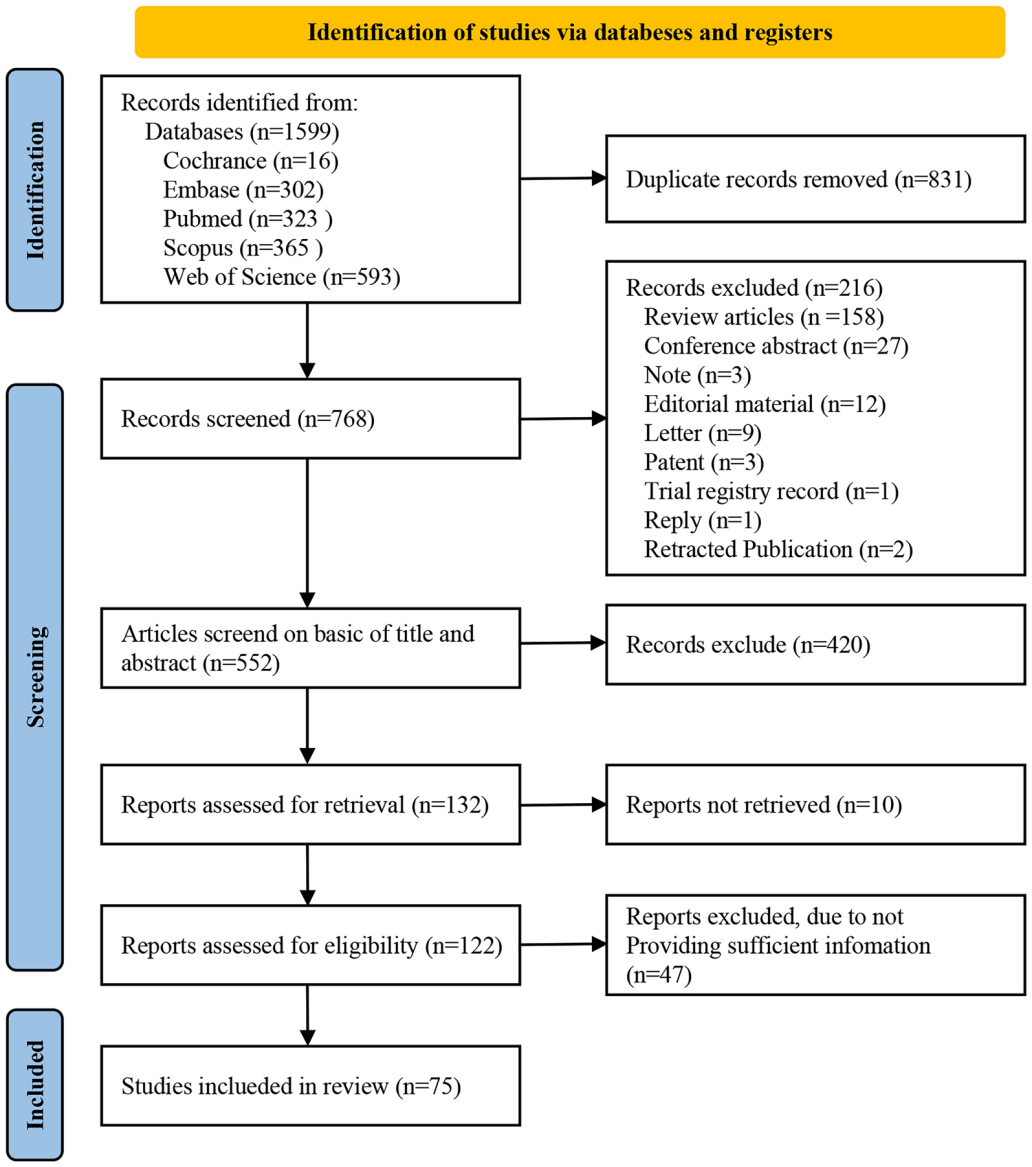
Flow diagram of the study selection

According to the PRISMA guidelines for study selection and exclusion, a total of 1599 records were identified during the search. Among them, 323 were from PubMed, 593 from Web of Science, 302 from Embase, 365 from Scopus, and 16 from Cochrane. After removing duplicates, there were 831 articles utilised for title and abstract analysis. After an initial screening, 158 reviews, 27 conference abstracts, 3 notes, 12 editorial materials, 9 letters, 3 patents, 1trial registry record, 1 reply and 2 retracted publications were excluded due to the type of study. A further 420 records were excluded after a critical analysis on the title and abstracts, leaving 132 articles for full-text retrieval. Of them, 10 articles were not accessible for retrieval. The full texts of 122 articles were accessed and assessed for eligibility, resulting in the inclusion of 75 articles in this study (Fig. 1).

## Oleanolic acid

### Physical and chemical properties

**Fig. 2:**
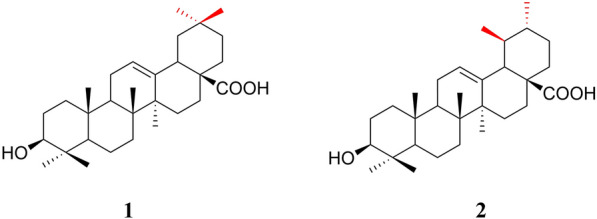
Chemical structures of oleanolic acid (OA [1]) and ursolic acid (UA [2])

OA (C_30_H_48_O_3_) is a commonly occurring pentacyclic triterpenoid compound found in both free acid and glycoside forms. It is often present alongside its structural isomer, UA in various plants. The main difference between OA and UA is the position of the methyl group on the E-ring (Fig. 2). OA, with a molecular weight of 456.7, is a hydrophobic compound, pale yellow, and non-volatile. It is nearly insoluble in water, sparingly soluble in ethanol and acetone, and soluble in 1-butanol, and its solubility increases with temperature [25]. OA exhibits a positive result in the Liebermann–Burchard test, indicating its triterpenoid nature. It does not show any reaction in the Molisch test, confirming the absence of connected glucose. Furthermore, when reacted with acetic anhydride–pyridine, OA forms acetyl esters, providing evidence that there are hydroxyl groups exist in the molecule [26].

### Botanical sources

OA is one of the most common pentacyclic triterpenoid compounds found in nature. It is widely distributed in the fruits, peels, leaves, and roots of various plants. Research has revealed that triterpenoid compounds are often concentrated in the wax layer of the plant epidermis, potentially attributed to their hydrophobic nature [27]. Olives are the most important source of OA, and it has been reported that the triterpene content of different varieties of virgin olive oils can be as high as 127–197 mg/kg, with OA being the most abundant, with an average content of 17.75 mg/kg [28]. Fructus Ligustri Lucidi, a traditional Chinese medicine, is also a rich source of OA that is widely used to treat various diseases [29]. Furthermore, OA has been detected in several common fruits such as apple [30], loquat [31], grape [32], and pomegranate [33]. Other edible medicinal plants, including ginseng [34], mistletoe [32], papaya [35], and hawthorn [36] also contain OA. Furthermore, a significant amount of OA is present in various herbs such as rosemary, thyme, and lavender [32]. OA can also be found in the bark of certain plant species, such as *Eucalyptus globulus* (southern blue gum tree) [37].

### Pharmacology

**Fig. 3:**
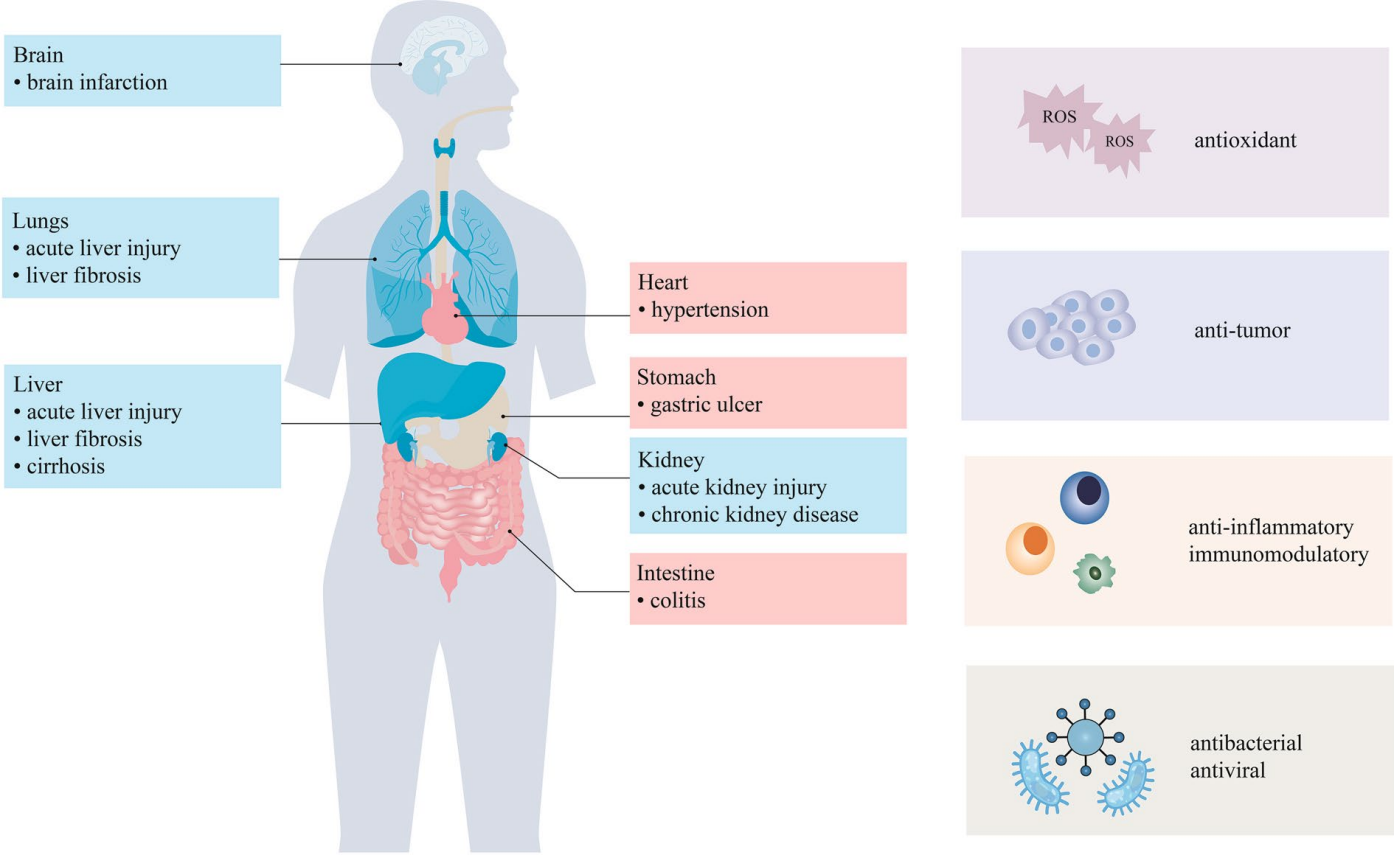
Potential pharmacological effects of oleanolic acid (OA)

OA exhibits a wide range of pharmacological effects and exerts protective effects in the whole body (Fig. 3). Hepatoprotection is among its most significant pharmacological actions. Multiple studies have demonstrated the hepatoprotective effects of OA against acute liver injury, as well as its potential for alleviating liver fibrosis and cirrhosis. Furthermore, OA has been found to induce apoptosis in liver cancer cells [38–40]. Presently, OA is classified as an over-the-counter drug in China for the treatment of' acute and chronic hepatitis [41]. Various experimental models have also confirmed the cardioprotective effects of OA. For instance, OA can prevent dexamethasone-induced hypertension [42] and regulate the release of prostacyclin (PGI2) from human coronary artery smooth muscle cells to maintain vascular homeostasis [43]. Ischemic stroke is the leading cause of mortality in humans [44]. OA has shown potential in alleviating brain infarction in a transient middle cerebral artery occlusion mouse model following reperfusion, and it can also improve chronic brain damage caused by ischemic stroke [45]. Studies have also reported that OA can improve the inflammatory response and oxidative damage in streptozotocin (STZ)-induced diabetic rats [46]. Recent studies have reported that OA exhibits protective effects on the gastrointestinal tract. Treatment with OA in acetic acid-induced chronic gastric ulcer model rats resulted in a reduction in lesion area and an increase in mucosal thickness, promoting gastric wound healing [47]. Additionally, OA can prevent acute lung injury induced by peroxynitrite and exerts a significant protective effect on pulmonary fibrosis [48, 49]. Another study demonstrated that OA can ameliorate dextran sulphate sodium-induced colitis [50]. In recent years, research has focused on the protective effect against kidney disease. Pre-treatment with OA improves renal injury induced by ischemia/reperfusion (I/R), as well as kidney fibrosis in a unilateral ureteral obstruction (UUO) mouse model [18, 51]. Furthermore, OA has been found to induce apoptosis in various types of tumour cells, including human hepatocellular carcinoma cells [39], breast cancer cells MCF-7 and MDA-MB-231 [52], and non-small cell lung cancer cells [53]. Increasing evidence suggests that OA possesses various biological activities, including anti-inflammatory, antioxidant, immunomodulatory, antibacterial, and antiviral [54–58].

### Pharmacokinetics

Pharmacokinetics is the study of the body's role in drug regulation, employing kinetic principles and mathematical models to quantitatively describe drug absorption, distribution, metabolism, and excretion [59]. Pharmacokinetic research is crucial in the context of novel drug development. The Caco-2 cell model is commonly used as an in-vitro absorption-prediction model, with experiments conducted using its cell monolayers to evaluate the absorption properties of OA [60]. The obtained apparent permeability coefficient (P_APP_) values for OA at concentrations of 10 and 20 μM were found to range from 1.1–1.3 × 10^−6^ cm/s, closely resembling the P_APP_ value of the low-permeability standard drug, atenolol (0.25 × 10^−6^ cm/s). Consequently, these results indicate a potential limitation in the absorption capacity of OA. Furthermore, Jeong et al. [60] found no significant difference between the apical-to-basolateral P_APP_ and basolateral-to-apical P_APP_, indicating that the transintestinal barrier transport of OA occurs via passive diffusion. Subsequently, researchers conducted extensive pharmacokinetic studies of OA in both animals and humans (Table 1).The study revealed that OA has a relatively short elimination half-life and very low absolute bioavailability (only 0.7 in rats) [60]. In addition to plasma, OA is widely distributed in tissues, with detectable levels found in the brain, heart, liver, kidneys, colon, and bladder of animals [61]. These data suggest that the very low bioavailability of OA may be attributed to its rapid clearance rate and extensive tissue distribution. Of note, OA is most extensively distributed in the liver, consistent with its established hepatoprotective effects. However, on the other hand, long-term high-dose administration can lead to liver toxicity and bile stasis [62]. This suggests that a balance between the beneficial effects and potential toxicity of OA should be considered when using it clinically. Following coincubation of OA with rat liver microsomes in the presence of nicotinamide adenine dinucleotide phosphate (NADPH), a notable decrease of 60% in peak current was observed, indicating that OA may be metabolised in the rat liver. Subsequent testing of the cultures initially identified the major metabolites of OA as hydroxy- and dihydroxy-OAs [60]. When studying excretion, the minimal levels of OA detected in urine suggest that OA is primarily non-renal in its elimination [60]. However, screening of its phase II metabolites revealed that OA is excreted in urine in the form of sulphate and glucuronide conjugates [63], explaining why intact OA is not detected in urine. In summary, OA, classified as a Class IV compound in the biopharmaceutics classification system due to its low water solubility, poor apparent permeability, and extremely low bioavailability, is somewhat limited in its clinical utility [64].

**Table 1:** Pharmacokinetic parameters of OA in different formulations

Form	Species	Administration	Dose	AUC	C_max_	T_max_	t1/2	CL	References
Capsule	Human	Oral	40 mg/kg	124.29 ng/h/mL	12.12 ng/mL	5.2 h	8.73 h	555.3 L/h	[152]
OA solution	Rats	i.v. injection	0.5 mg/kg 1 mg/kg 2 mg/kg	16 μg min/mL 32.6 μg min/mL 71.6 μg min/mL	NA	NA	41.9 min 52.7 min 48.0 min	31.7 mL/min/kg 33 ml/min/kg 28 mL/min/kg	[60]
OA solution	Rats	Oral	25 mg/kg 50 mg/kg	5.9 μg min/mL 10.7 μg min/mL	NA	25 min 21 min	46.5 min 65.3 min	NA	[60]
OA in olive oil	Human	Oral	30 mg	3181.9 ng h/mL	598.2 ng/mL	3.0 h	4.61 h	35.1L/h	[153]
OA into a metered-dose inhaler	Rats	Inhale	120 μg/mL	428.25 ng/h/mL	22.75 ng/mL	4 h	8.93 h	NA	[65]

Values are expressed as the mean ± standard error of the mean when available

AUC: area under the curve; C_max_: maximum plasma concentration; T_max_: time to maximum concentration; t1/2: half-life; CL: clearance; NA: non-available data

### Oleanolic acid derivatives

**Fig. 4:**
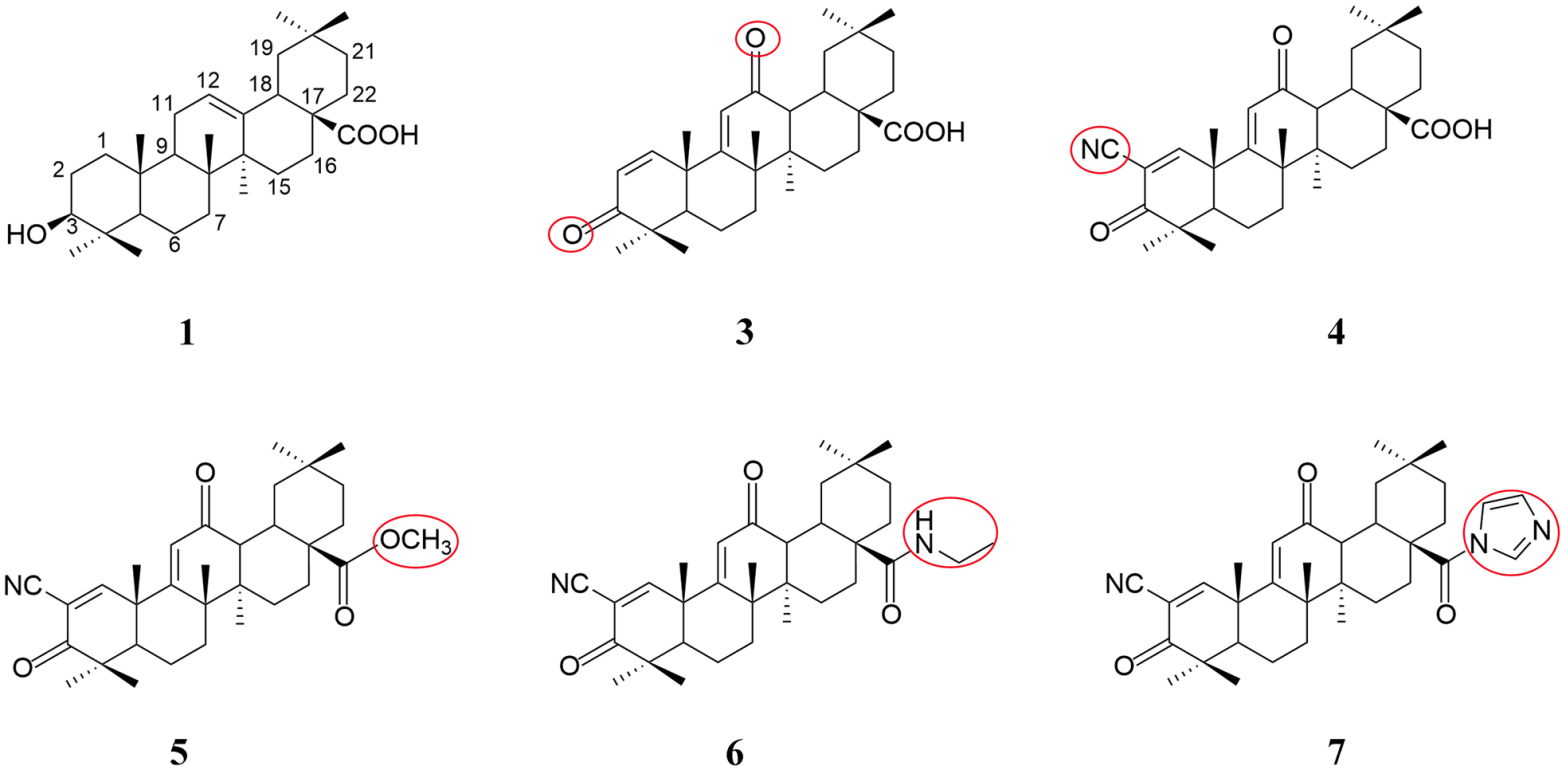
Oleanolic acid (OA [1]) and its derivatives

OA exhibits a wide range of pharmacological activities, however, it has poor water solubility and low bioavailability. Extensive research has been conducted to enhance its bioavailability, including modifications to OA and the design of a series of derivatives, which have demonstrated potent biological activities such as antitumour, antioxidant and anti-inflammatory effects [66–68]. In 1998, approximately 60 OA derivatives were synthesised to screen for compounds capable of inhibiting the production of nitric oxide (NO) in macrophages. These derivatives were subsequently tested in vitro, and nine were found to exhibit substantial inhibition of INF-γ-induced NO production in macrophages. Among these derivatives, 3,12-dioxoolean-1,9-dien-28-oic acid (Fig. 4(3)) showed the highest inhibitory activity [69]. Based on these findings, a more potent derivative, 2-cyano-3,12-dioxooleana-1,9-dien-28-oic acid (CDDO) (Fig. 4(4)), was synthesised by introducing an electron-withdrawing cyano group at the C-2 position. This compound exhibited an inhibitory effect on NO that was 400 times stronger than the previously synthesized compounds [70]. CDDO-methyl ester (CDDO-Me) (Fig. 4(5)) is CDDO modified by methyl esterification, and this compound not only possesses potent anti-inflammatory activity and antioxidant activity [71]. CDDO-Ethyl Amide (CDDO-EA) (Fig. 4(6)) involves the introduction of an ethyl amide group at the C-17 position of CDDO [72]. Similarly, utilizing CDDO as the precursor material, CDDO-imidazole (CDDO-Im) (Fig. 4(7)) is synthesised by introducing an imidazole ring at the C-28 position [72]. Currently, both of these compounds demonstrate promising anti-cancer effects, exhibiting inhibitory effects on various cancer cells [73–76]. Among them, CDDO series derivatives have shown remarkable therapeutic effects in renal diseases. Pre-treatment with CDDO-Me has been shown to mitigate AKI induced by I/R in rats via its anti-inflammatory, antioxidant, and anti-apoptotic properties [77]. Additionally, CDDO-Im activates the Nrf2 signalling pathway, conferring protective effects against bilateral ischemic AKI in mice [78]. Moreover, CDDO-Me has demonstrated the ability to ameliorate mouse renal fibrosis mediated by aristolochic acid [79].

### Safety

OA is a relatively safe natural compound. In an early brine shrimp test, the median lethal concentration (LC50) of OA was determined to be 0.95 mg/mL [80]. A study investigating the acute and chronic toxicity of a mixture of UA and OA reported that single subcutaneous injections of 300 mg/kg in Balb/c mice, which were then observed for 14 days, along with repeated injections of 6.5 mg/kg and 13 mg/kg of UA/OA within 28 days, did not result in any notable alterations or abnormalities in the biochemical indicators or major organ histopathological examinations when compared to the control group. The median lethal dose (LD50) was determined to be greater than 300 mg/kg [81]. Furthermore, the effects of administrating a single dose of 120 μg of UA and 120 μg of OA via a metered dose inhaler in rats were monitored, and the results revealed no signs of clinical toxicity [65]. A clinical trial involving 70 cases of acute hepatitis demonstrated that continuous daily administration of 60–90 mg of OA for 30 days showed no significant adverse effects and exhibited therapeutic efficacy [82].

## Role of OA in kidney disease

Currently, research on the therapeutic potentials of OA, UA, and their derivatives in relation to kidney diseases primarily use animal and cellular models, with a specific focus on acute kidney injury and chronic kidney disease.

### AKI

Research has indicated that the pathogenesis of AKI is related to several factors, including renal ischemia, nephrotoxins, sepsis, infections, contrast agents, and drug induction [83–85]. In recent years, the application of OA and its analogues as treatment methods for AKI has received considerable attention. OA has been shown to exhibit preventive effects against the occurrence and progression of AKI through various mechanisms, including protection against anti-oxidative stress, inhibition of inflammatory responses, enhancement of autophagy, and exhibiting anti-apoptotic properties [77, 86–88]. Furthermore, compounds related to OA have been found to modulate multiple signalling pathways, thereby improving the renal damage caused by AKI and restoring renal tubular function. For example, UA exerts its protective effects on renal I/R injury in mice by modulating the NF-κB pathway and inhibiting the production of inflammatory factors such as IL-1β, IL-6, TNF-a, and ICAM [89]. Pretreatment with OA can activate the antioxidant factor Nrf2; regulate GCLc; reduce the serum malondialdehyde levels; enhance the activities of SOD, catalase, and glutathione; and alleviate oxidative damage [18]. Additionally, UA can enhance macrophage autophagy and suppress the inflammatory response in LPS-induced AKI mice by modulating the toll-like receptor (TLR)4/MyD88 pathway [88].

### CKD

CKD is typically a progressive disease characterised by the gradual deterioration of kidney function, and it is caused by a range of diseases, including diabetes, hypertension, AKI, glomerulonephritis, and obesity [90–92]. Increasing evidence suggests that OA and its analogues exert a protective effect against CKD. Clinical studies have demonstrated that the CDDO-Me can increase the estimated GFR in patients with moderate to severe CKD and type 2 diabetes, while reducing serum creatinine (Scr) and blood urea nitrogen (BUN) levels, indicating that these patients experience a short-term improvement in renal function [93]. Renal interstitial fibrosis is a key characteristic of CKD. Studies have shown that OA regulates the abnormal accumulation of extracellular matrix through the transforming growth factor-β1 (TGF-β1)/Smad pathway, thereby alleviating renal fibrosis in a mouse model for UUO [94]. Furthermore, UA has demonstrated the ability to reduce the expression of inflammatory factors, diminish the excessive accumulation of extracellular matrix, and improve renal injury in db/db mice [95].

The aforementioned evidence suggests that OA-related compounds exert a protective effect against renal diseases. However, the specific mechanisms underlying their effectiveness remain poorly understood. In the following sections, we provide a detailed overview of the mechanisms through which OA and its analogues exert their effects on kidney disease, both in vitro and in vivo. The foundational studies investigating the effects of OA, its isomer UA, and its derivatives on kidney disease, mainly involving disease models, intervention methods, and underlying mechanisms have been summarized in Table 2.

**Table 2:** Effects and mechanisms of oleanolic acid (OA) and its analogues for the treatment of kidney disease

Type of study	Cell/animal model	Drug and dose	Targets	References
In vivo	APAP-induced renal damage in Wistar rats	OA (5, 25 mg/kg)	NO↓, GSH↑	[86]
In vivo	STZ-induced DN in SD rats	OA (60 mg/kg)	MAP↓	[147]
In vivo	STZ-induced diabetic C57BL/6 mice with BALB/c islets	OA (0.5 mg/d)	IL-10↑, VEGF↑, IFN-γ↓, IL-4↓, IL-17↓, IL-2↓, CD4+ ↓, CD8+ ↓	[110]
In vivo	STZ-induced DN in Balb/cA mice	OA or UA (0.05%, 0.1%, 0.2% in food)	AR↓, SDH↓, GLI↑	[146]
In vivo	STZ-induced DN in C57BL mice	UA (0.01% in food)	ColIV↓, pSTAT3↓, iNOS↓, p-JNK↓, p-ERK↓	[154]
In vivo	IR in C57BL/6 mice	BARD (20 mg/kg)	Nrf2↑, PPAR-γ↑, HO-1↑	[155]
In vivo	STZ-induced DN in SD rats	OA (30, 60, 120 mg/kg)	Urinary Na^+^ output↑	[22]
In vivo	STZ-induced diabetic C57BL/6 mice with BALB/c islets	OA (25 mg/kg)	IL-10↑, VEGF↑, IFN-γ↓, IL-1β↓, IL-4 ↓, IL-17↓, IL-2↓, CD4+ ↓, CD8+ ↓	[156]
In vivo	STZ-induced DN in SD rats	OA (20, 40, 80 mg/kg)	SOD↑, catalase↑	[157]
In vivo	STZ-induced DN in Wistar rats	UA (0.2% in food)	8-OHdG↓, NF-κB↓	[158]
In vivo	UUO in C57BL/6 mice	OA (60 mg/kg)	Bax/Bcl2↓, Nrf2↑, HO1↑, Hsp70↑, NAD(P)H↑	[51]
In vivo	CsA-induced kidney injury in ICR mice	OA (25 mg/kg)	8-OHdG↓, 8-iso-PGF2α↓, nuclear/total Nrf2↑, HO-1↑, Bax/Bcl-2↓	[125]
In vivo In vitro	Bilateral ischemic AKI in wild-type mice glucose/serum starvation-induced in renal epithelial cell	CDDO-Im (30 μmol/kg)	IL-6↓, G-CSF↓, KC↓, Nrf2↑, HO-1↑, Nqo1↑, Gclc↑	[78]
In vivo	CCl_4_-induced oxidative damage in ICR mice	UA (25, 50 mg/kg)	ROS↓, 8-OHdG↓, TNF-α↓, IL-6↓, IL-17↓, COX-2↓, p-STAT↓, nuclear/total NF-κB↓	[100]
In vivo	(AA)-induced acute kidney injury in C57BL/6 mice	BARD (10 mg/kg)	Nrf2↑, Keapl↓, HO-1↑, NQO1↑, ROS↓	[159]
In vivo	(AA)-induced acute kidney injury in zebrafish	UA (10 ppm)	TNF-α↓, mpo↓	[160]
In vitro	Rat mesangial cells cultured under HG conditions	UA (2.5 μM)	miRNA21↓, P85PI3K↓, pAkt↓, pmTOR↓, p62/SQSTMI↓, COL-I↓, LC3II↑, PTEN↑	[137]
In vitro	Podocytes cultured under HG conditions	UA (1, 2.5, 5, 7.5 μM)	Synaptopodin↑, podocin↑, nephrin↑, miRNA-21↓, PI3K↓, p-Akt↓, p-mTOR↓, Beclin1↑, p62↓, COL-I↓, LC3II/LCI↑, PTEN↑	[138]
In vivo	IR in Wistar rats	CDDO-Me (20mg/kg)	TAS↑, TOS↓, OSI↓, TT↑, NO↓, ADMA↓, SOD↑, GSH-PX↑, iNOS↓, PPAR-ɣ↑, Nrf2↑, NF-KB↓	[77]
In vivo	OLETF (type 2 diabetes mellitus model) rats	OA (100 mg/kg/day) OA (5 μM)	Nephrin↑, VEGF↓, ESAM↑, TGF-β↓, p-smad2/3↓, p-ERK↓, p-eIF2α↓, ATF6↓, Bip↓, CHOP↓, Bcl2↑, Bax↓, Caspase 3↓, Nrf2↑, HO-1↑, α-SMA↓	[124]
In vivo	IR in Wistar rats	OA (12.5, 25, 50 mg/kg)	KIM-1↓, MDA↓, SOD↑, CAT↑, GPx↑, GSH↑, IFN-γ↓, IL-6↓, IL-10↑, MPO↓, Caspase 3↓, Nrf2↑, GCLc↑	[18]
In vivo	IR in SD rats	UA (10 mg/kg)	TNF-α↓, IL-1β↓, IL-6↓, IL-10↑, p-STAT3↓, P65↓, Caspase-3↓	[101]
In vivo	STZ-induced DN in SD rats	UA (20 mg/kg)	GR enzyme↓, MDA↓, GSH↑, CAT↑, SOD↑	[161]
In vitro	NRK-52E cell stimulated with TGF-β1	OA (2, 4, 8 μM)	E-cadherin↑, FN↓,α-SMA↓, Nrf2↑, klotho↑, p-Smad2/3↓, ILK↓, Snail↓	[133]
In vivo	STZ-induced DN in Wistar rats	UA (25 mg/kg)	TNF-α↓, IL-1β↓, IL-6↓, IL-18↓, TLR4↓,NF-κB↓, MyD88↓	[105]
In vivo	Adenine-induced CKD in Wistar rats	UA (30 mg/kg)	TGF-β↓, CTGF↓, FN↓, CoL-I↓	[162]
In vivo	STZ-induced DN in SDrats	UA (35 mg/kg)	SOD ↑, MDA↓,TNF-α↓, MCP-1↓, IL-1β↓	[163]
In vivo	CLP in mice	UA (2, 20 mg/kg)	ROS↓, 8-OHdG↓, SOD1↑, SOD2↑, TNF-α↓, IL-1β, IL-6↓, NF-κB↓	[164]
In vitro	NRK-52E cell stimulated with HG	OA (10 μM)	miR-142-5p↓, PTEN↑, p62↓, LC3-II/ LC3-I↑, a-SMA↓, CoL-IV↓, PI3K↓, pAKT↓, p-mTOR↓	[21]
In vitro	HEK293T cells stimulated with OTA	UA (1 μM)	ROS↓, Lonp1↑, Aco2↑, Hsp75↑	[165]
In vivo In vitro	db/db Mice Mesangial cells stimulated with HG	UA (0.3% in food) UA (2.5 μM)	ARAP1↓, AT1R↓ 8-OHdG↓, NOX2↓, FN↓, IL1β↓, IL-18↓, TGF-β1↓, CoL-IV↓	[95]
In vivo	Cisplatin-induced nephrotoxicity in mice	OA (10, 40 mg/kg)	HO-1↓, SOD↑, 4-HNE↓, TNF-α↓, p-STAT3↓, NF-κB↓	[102]
In vivo In vitro	UUO in C57BL6 mice HK-2 cells stimulated with TGF-β1	UA (50 and 100 mg/kg) UA (10, 50 μM)	CoL-I↓, FN↓, a-SMA↓, E-cadherin↓, p-Smad3↓	[132]
In vivo In vitro	LPS-induced AKI in BALB/C mice RAW 264.7 stimulated with LPS	UA (100 mg/kg) UA (10 μM)	TNF-α↓, IL-6 ↓, IL-1β↓, F4/80↓, TLR4↓, MyD88↓, LC3B↑, Beclin-1↑	[88]
In vivo	Pristane-induced lupus nephritis in BALB/c mice	AOA (50 mg/kg)	RORγt↓, IL-17A↓, IL-17F↓, IL-22↓, IFN-γ↓, IgG↓,IgM↓, Th17↓	[109]
In vivo	UUO in SD rats	OA (6 mg/kg)	CoL-I↓, CoL-III↓, FN↓, α-SMA↓, TGF-β↓, TβRI↓, TβRII↓,p-Smad2↓	[131]
In vitro	Podocyte stimulated with sC5b‐9	OA (10, 20, 40, 80, 160 µM)	nephrin↑, podocin↑, CD2AP↑, Bcl2↑, Bax↓, p‐AKT↓, p‐mTOR↓	[166]
In vivo	Pristane-induced lupus model in BALB/c mice	OA (50 mg/kg)	Th17↓, IL-17A↓, IFN-γ↓, IgG↓, IgM↓	[20]
In vivo In vitro	COM-induced kidney damage in SD rats HK-2 cells stimulated with COM	UA (20, 40 mg/kg) UA (2.5, 5 μM)	α-SMA↓, CoL-I ↓, Bcl-2↑, Bax↓, Nrf2↑, HO-1↑, TNF-α↓, IL1β↓, IL-6↓, TLR4↓, p-NF-κB↓	[104]
In vivo	CP-induced nephrotoxicity in Wistar rats	UA (5, 10 mg/kg)	GSH↑, SOD↑, CAT↑, MDA↓, IL-1β↓, IL-6↓, TNF-α↓, Caspase-3↓, Caspase-9↓	[141]
In vivo	STZ-induced DN in SD rats	UA (50 mg/kg)	TNF-α↓, IL-1β↓, IL-6↓, SOD↑, MDA↓, GSH↑, CAT↑, NO↓, FN↓, E-cad↑, MMP-9↑,TIMP-1↓, α-SMA↓, TGF-β1↓,SMA3↓, SMA7↓, P38↓	[167]
In vivo	RIRI in SD rats	OA (50 mg/kg)	PI3K↓, p-AKT↓, PDK1↑, p27↑, TRAP1↑, CypD↓	[168]
In vitro	HK-2 cells stimulated with OTA	OA (2 μM)	Bax↓, Bcl-2↑, Cyt-C↓, Caspase-9↓,Caspase-3↓, GRP78↓, CHOP↓	[169]
In vivo	STZ-induced DN in SD rats	OA (50, 100 mg/kg)	nephrin↑, CD68↓, COL-IV↓, p-AMPK/AMPK↑, PGC-1α↑, TLR4↓, NF-κB↓, TGF-β1↓	[103]
In vivo	STZ-induced DN in SD rats	OA (50 mg/kg)	Caspase-3↓, Bax↓, CD31, E-cadherin↑, α-SMA↓, Vimentin↓, TGF-β1↓, p-P38↓, FGFR1↑, SIRT3↑, DPP-4↓	[170]
In vivo	UUO in SD rats	UA (40 mg/kg)	TGF-β1↓, Keap1↓, Nrf2↑, HO-1↑, 8-oxo-dG↓, Caspase-3↓, Caspase-8↓	[171]
In vivo	TAA-induced AKI in BALB/c mice	OA (45, 90 mg/kg)	MDA ↓, NOx ↓, GSH↑, SOD↑, NF-κB↓, TNF-α↓, Bax↓, Bcl2 ↑, Caspase-3↓ Nrf2↑, HO-1↑	[143]
In vitro	HK-2 cells stimulated with OTA	UA (4 μM)	Lonp1↑, Sig-1R↑, GRP78↓, p-ERK↓, p-eIF2α↓, CHOP↓, IRE1α↓, Bcl2↑, Bax↓	[142]

(↑) and (↓) signs show positive and negative effects of oleanolic acid and its analogues on its target molecules, respectively

## Specific mechanisms of action of OA in relation to kidney disease

### Anti-inflammatory

The renal inflammatory response serves as an initial protective reaction against kidney damage. However, unresolved inflammation can recruit additional immune cells and trigger the production of a cascade of proinflammatory factors [96]. Consequently, intrinsic renal cells are activated, leading to the release of profibrotic factors such as α-smooth muscle actin (α-SMA), which promotes renal fibrosis and further exacerbates kidney injury [97]. An increasing body of evidence suggests that OA and its analogues possess significant anti-inflammatory effects by modulating various inflammation-related pathways, including nuclear factor-κB (NF-κB), signal transducer and activator of transcription (STAT), and Toll-like receptor 4 (TLR4) [98, 99].

In a mouse model of carbon tetrachloride (CCl_4_)-induced renal injury, UA significantly reduces the production of proinflammatory cytokines such as tumor necrosis factor-alpha (TNF-α), interleukin (IL)-6, and IL-17. Subsequent studies have reported that CCl_4_ treatment increases the phosphorylation of STAT3 (p-STAT3) and activates the NF-κB pathway, whereas UA treatment inhibits this process. Therefore, the inhibitory effect of UA on inflammatory responses may thus be associated with the modulation of the STAT3 and NF-κB pathways [100]. Additionally, pretreatment with UA has been shown to significantly improve acute kidney injury induced by IR in rats. It reduces the expression of TNF-α, IL-6, IL-1β, STAT3 and NF-κB [101]. Similar effects were observed in a mouse model of cisplatin-induced nephrotoxicity treated with OA [102]. Another study revealed that OA interventions significantly alleviated renal injury in a rat model for diabetic nephropathy (DN). It reduced the expression of macrophage marker CD68 and decreased the relative protein expression levels of TLR4 and nuclear NF-κB [103]. Concurrently, stimulation of HK-2 cells with calcium oxalate monohydrate (COM) crystals significantly increased the expression of TNF-α, IL-1β, and IL-6, and elevated levels of TLR4 and p-NF-κB proteins. However, treatment with UA inhibited this process, suggesting that UA may suppress inflammation through the TLR4/NF-κB pathway [104]. Li et al. found that UA reduced the levels of serum TNF-α, IL-6, IL-1β, and IL-18 in DN rats. It also inhibited the expression of TLR4, myeloid differentiation primary response gene 88 (MyD88), and NF-κB proteins [105]. In conclusion, OA-related compounds may inhibit inflammatory responses and attenuate renal damage via the TLR4/NF-κB signaling pathway.

### Immunoregulatory effect

The immune system plays a crucial role in renal diseases. Under steady-state conditions, the kidney harbours immune cells, including macrophages, dendritic cells, and lymphocytes. When the kidneys are damaged, these immune cells release a range of proinflammatory mediators, such as chemokines and cytokines, which further promote the occurrence and progression of inflammation [106]. Furthermore, immune system dysregulation can give rise to the occurrence of autoimmune kidney diseases, including lupus nephritis and IgA nephropathy, which can inflict severe damage upon the kidneys [107].

Lupus nephritis is an immune complex nephritis. T helper cell (Th17) plays an important role in the pathogenesis of lupus nephritis [108]. Research has demonstrated that, in mice with pristane-induced lupus nephritis, treatment with OA reduces dsDNA levels, IL-17A expression and interferon γ (IFN-γ), and alleviate renal injury by decreasing the deposition of IgG and IgM in the glomeruli [20]. Further studies have revealed that OA inhibits the differentiation of Th17 cells in vitro. Additionally, Zhou et al. found that 3β-acetoxy-oleanolic acid (AOA) exhibits similar beneficial effects in an LN model [109]. Furthermore, OA has been shown to have a preventive effect on immune responses caused by pancreatic islet transplantation. It decreases the expression of IFN-γ, IL-4, IL-2, and IL-17 in T cell populations while markedly increasing the levels of IL-10. OA also reduces the infiltration of CD4+ and CD8+ T cells and extends the survival time [110]. Likewise, in a model where kidneys from BN rats were transplanted into LEW rats, the combined treatment of OA and cyclosporine A decreased the infiltration of CD4+ and CD8+ T cells and ameliorate rejection and inflammatory responses following kidney transplantation in rats [111]. In our investigation, we discovered that human monocytes and mouse macrophages displayed an exaggerated sensitivity to monocyte chemoattractant protein-1 when subjected to modulation by high glucose (HG) and human low-density lipoprotein. Notably, this heightened response was effectively mitigated by UA [112]. Furthermore, the administration of UA in the LPS-induced AKI model reduces the infiltration of F4/80-positive macrophages and a decrease in the expression of proinflammatory cytokines, including TNF-α, IL-6, and IL-β. However, UA demonstrated the ability to decrease the expression levels of TNF-α, IL-6, and IL-1β in RAW264.7 cells stimulated with LPS [88].

### Improving oxidative stress

Oxidative stress (OS) refers to the imbalance between oxidation and antioxidant mechanisms in the body, leading to the excessive production of reactive oxygen species (ROS) and reactive nitrogen species (RNS) or insufficient antioxidant defence mechanisms [113, 114]. The kidney is a highly oxygen-consuming organ, second only to the heart, and is thus particularly vulnerable to OS-induced damage [115]. There is growing evidence that OS plays a crucial role as a pathological driving factor in the occurrence and progression of kidney disease. This includes the induction of damage to proximal tubular epithelial cells, resulting in cell death and impairment of proximal tubular reabsorption [116]. Early clinical studies have indicated that patients with sepsis-induced AKI exhibit mitochondrial dysfunction and excessive NO production [117]. Furthermore, OS levels gradually increase with the progression of CKD and are significantly correlated with the level of renal function [118]. OA is an effective antioxidant that directly reacts with ROS, scavenges free radicals, and enhances antioxidant defence. It promotes antioxidant glutathione generation via nuclear factor erythroid 2-related factor 2 (Nrf2) and increases the expression of antioxidant enzymes [119]. Simultaneously, CDDO-Me synthesised from OA as a precursor compound shows stronger antioxidant capacity and is the most effective activator of Nrf2 pathway [120, 121], which can improve the estimated GFR, reduce the urinary albumin-to-creatinine ratio, and restore renal function in patients with moderate to severe CKD [122, 123].

HG-induced OS is believed to be a primary cause of diabetic renal fibrosis. In a 20-week study involving OLETF (type 2 diabetes model) rats, continuous administration of OA resulted in a decrease in the expression of 8-hydroxy-2′-deoxyguanosine (8-OHdG), which is a marker of oxidative DNA damage. Furthermore, the levels of the antioxidant enzyme superoxide dismutase (SOD) were also elevated. Subsequent in-vitro experiments involving stimulation of mouse mesangial cells with high glucose and toxic β-carotene induced endoplasmic reticulum stress, leading to elevated levels of ROS. Results indicate that similar to the action of the endoplasmic reticulum stress inhibitor 4-phenylbutyric acid, OA can reverse this effect. These findings suggest that OA administration can effectively reduce oxidative damage and promote renal repair [124]. Nrf2 is a critical transcription factor that binds to antioxidant response elements and regulates antioxidant responses under OS conditions. In a mouse model of UUO, treatment with OA was observed to decrease renal tubular injury and increase Nrf2, while the expression of Kelch-like ECH-associated protein 1 (Keap1) remained unchanged. These findings indicate that OA facilitates the nuclear translocation of Nrf2, leading to a reduction in renal OS [51]. Similarly, OA intervention markedly increased the nuclear Nrf2/total Nrf2 ratio, as well as the levels of heme oxygenase-1 (HO-1) and SOD in mice with chronic cyclosporine nephropathy. Furthermore, OA treatment reduced the levels of malondialdehyde, a marker of OS [125]. Moreover, pretreatment with OA has been found to decrease NO levels in the kidneys of rats with acetaminophen-induced liver and kidney toxicity. It also elevates the intracellular levels of glutathione in renal cells, leading to the mitigation of liver and kidney injury [86]. Treatment of HK-2 cells exposed to cisplatin with CDDO-Me upregulates mRNA expression of HO-1, NAD(P)H, glutathione peroxidase 1, and catalase, along with other antioxidant enzymes. This upregulation effectively prevents cellular senescence [126].

### Anti-fibrosis

Renal fibrosis is a common outcome of many chronic and progressive kidney diseases, characterised by the deposition of extracellular matrix proteins, notably collagen and adhesive glycoproteins such as fibronectin (FN) [127]. Activated myofibroblasts are considered important cells involved in the secretion of these matrix components, including α-SMA which serves as a marker for myofibroblast activation and TGF-β1 which is the key regulatory factor in the modulation of renal fibrosis [128]. Abundant foundational research has demonstrated the potential of OA-related compounds in ameliorating fibrosis in multiple organs, including the heart, lungs, liver, and kidneys [40, 51, 129, 130]. Thus, targeting fibrosis may thus represent a potential therapeutic strategy for OA in the treatment of kidney diseases.

Treatment with OA demonstrates a significant reduction in Scr, BUN, and urinary protein levels in the UUO rat model, thereby mitigating renal collagen deposition. Moreover, OA administration downregulates the mRNA expression of collagen (COL)-I, COL-III, FN, and α-SMA in the kidneys. Additionally, OA decreases the protein levels of TGF-β, TGF-β receptor (TβR) I, TβRII, and phosphorylated Smad2/Smad2 (p-Smad2/Smad2). These results indicate that OA may ameliorate fibrosis in UUO rats by modulating the TGF-β/Smad signalling pathway [131]. Xu et al. also found that UA inhibits epithelial-mesenchymal transition (EMT) by reducing the protein expression of TGF-β1 and p-Smad3, thereby reducing renal fibrosis in the UUO model. Further research has shown that UA can decrease the expression of pro-fibrotic factors in TGF-β1-treated HK-2 cells, lower the protein levels of TGF-β1 and p-Smad3, and inhibit EMT [132]. Similarly, He et al. induced EMT with TGF-β1 and intervened using OA to culture renal tubular epithelial cells NRK-52E. The results demonstrated that TGF-β1 stimulation leads to the downregulation of the epithelial cell marker E-cadherin and the upregulation of α-SMA and FN expression; however, treatment with OA effectively inhibited this process along with the expression of p-Smad2/3, a downstream component of the TGF-β signalling pathway [133]. The aforementioned findings suggest that the inhibitory effects of OA and its analogues on renal fibrosis may be mediated through the TGF-β/Smad signalling pathway.

### Enhancing autophagy

Autophagy refers to the cellular process by which damaged organelles, proteins, and other cellular components are degraded through lysosomes, which helps maintain intracellular homeostasis while simultaneously generating energy [134]. Increasing evidence suggests that autophagy plays a protective role in renal diseases. In proximal tubular autophagy-deficient mice, following I/R injury, significant increases in Scr and BUN levels were observed when compared to those in wild-type control mice. Additionally, there was a marked accumulation of p62 and ubiquitin aggregates was observed, indicating prominent proximal tubular injury [135]. Furthermore, in the UUO rat model, continuous intraperitoneal injection of the autophagy inhibitor 3-methyladenine for 7 days exacerbated tubular epithelial cell apoptosis and interstitial fibrosis in the rat kidneys [136]. Chen et al. found that intervention with OA significantly alleviated renal fibrosis in a STZ-induced DN mouse model. OA treatment reduced macrophage infiltration and decreased the expression of α-SMA and CoL-IV. Furthermore, compared with those in the control group, the autophagy markers LC3I and LC3II were downregulated in the DN model, indicating that autophagy levels are suppressed during the progression of diabetic kidney disease. OA may exert a protective effect in diabetic nephropathy by enhancing autophagy [21].

Further studies have revealed revealed that miRNA is associated with autophagy in diabetic nephropathy, and the phosphatase and tensin homolog (PTEN) are the downstream target genes. An in vitro model of autophagy was established by subjecting NRK-52E cells to HG treatment. In the HG group, CoL-IV was upregulated, whereas LC3I and LC3II were downregulated. The expression of miR-142-5p was increased, and PTEN expression was decreased. These results were reversed by OA treatment. Furthermore, OA reduced the expression of PI3K, p-AKT, and p-mTOR proteins [21]. Similarly, in vitro cultures of rat glomerular mesangial cells and podocytes under HG conditions were were treated with UA. The deposition of collagen within the cells decreased, and transmission electron microscopy revealed suppressed autophagosome reduction, and accompanied by the downregulation of the autophagic lysosomal degradation marker p62. Additionally, the expression of LC3I, LC3II, and PTEN was increased, whereas that of miRNA-21 was suppressed, and the PI3K/AKT/mTOR pathway was inhibited [137, 138]. These results indicate that UA may enhance the level of intrinsic cellular autophagy in the kidneys through the PI3K/AKT/mTOR signalling pathway, thereby alleviating renal damage. Furthermore, in an LPS-induced macrophage model, UA was found to regulate the TLR4/MyD88 pathway and inhibit the release of inflammatory factors. This anti-inflammatory action of UA can be blocked by the autophagy inhibitor 3-methyladenine, suggesting that this action is mediated through autophagy [88].

### Others

#### Anti-apoptosis

Cell apoptosis refers to programmed cell death, which typically occurs during development and aging processes to help maintain cellular homeostasis within a population [139]. However, excessive apoptosis can deplete essential functional cells, leading to organ damage and even triggering inflammation [140]. UA has been reported to down-regulate the expression of IL-1β, IL-6 and TNF-α, reduce the levels of apoptosis markers caspase-3 and -9, and attenuate cisplatin-induced nephrotoxicity [141]. Treatment of HK-2 cells with ochratoxin A (OTA) enhances the expression of the proapoptotic gene Bax and inhibits the expression of the anti-apoptotic gene Bcl-2, leading to cell apoptosis, however, pretreatment with UA can alleviate this situation [142]. Furthermore, in a mouse model of liver and kidney injury induced by thioacetamide (TAA), OA can reduce the expression of Bax and caspase-3, increase the expression of Bcl-2, and alleviate liver and kidney damage [143]. Thus, the OA-related compounds may thus alleviate kidney injury through the anti-apoptotic pathway.

#### Anti-glycation

Protein glycation is a diverse post-translational modification of proteins that forms advanced glycation end products (AGEs), which can induce protein dysfunction and disrupt homeostasis [144, 145]. OA and UA treatment in diabetic mice has been shown to lower blood glucose levels and improve overall kidney function. To investigate whether their therapeutic effects on the kidneys are related to the anti-glycation properties of these two triterpenoid compounds, the enzymes involved in the polyol pathway of glucose metabolism and the levels of AGEs were examined. The results revealed that OA and UA were able to inhibit the activity of renal aldose reductase, enhance the activity of glyoxalase I, reduce the activity of sorbitol dehydrogenase, and suppress the generation of AGEs, such as plasma glycated haemoglobin, renal *N*-ε-(carboxymethyl)lysine, pentosidine, and urinary glycated albumin. These findings suggest that OA and UA may alleviate kidney damage by exerting anti-glycation effects [146].

#### Anti-hypertension

Hypertension is widely recognised as a primary catalyst of CKD progression and can significantly exacerbate renal injury [91]. In the STZ-induced diabetic rat model, OA was found to increase the sodium excretion rate without affecting the urine flow, K^+^, and Cl^−^ rates. Additionally, it decreased mean arterial pressure (MAP) in rats and increased creatinine clearance, leading to a reduction in the plasma creatinine concentration. These findings suggest that OA has a protective effect on renal function [147]. Studies have demonstrated that treatment with OA increases urinary Na^+^ excretion and decreases MAP in experimental models for hypertension. Furthermore, the reduction in MAP is directly proportional to the increase in urinary Na^+^ excretion. Based on these findings, OA is hypothesised to exert its renal protective effects by enhancing Na^+^ excretion, thereby lowering MAP [148].

## Limitations and optimization of OA for clinical applications

**Fig. 5:**
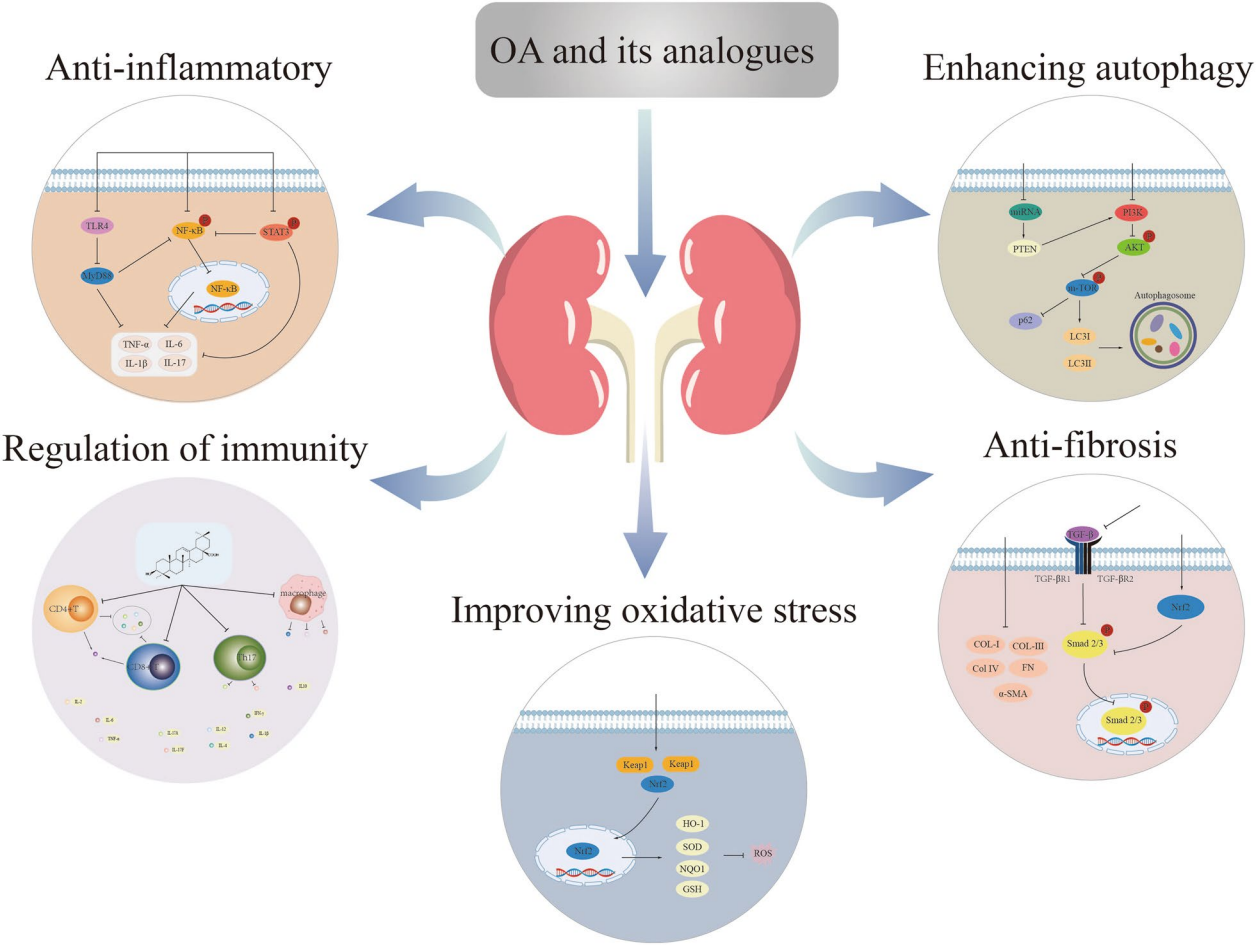
Main oleanolic acid mechanisms in relation to kidney disease

OA exhibits broad biological activity, and numerous studies have confirmed its protective effects on the kidneys. This review has summarized the known mechanisms of action of OA and its analogues in relation to kidney disease (Fig. 5). However, due to its unique chemical structure, poor water solubility, limited permeability, and low bioavailability, the clinical application of OA may be hindered. Therefore, improving the bioavailability of OA is thus a pressing issue that needs to be addressed in future research. A potential approach to address this issue is through the design and synthesis of derivatives using OA as a starting material. In this regard, 3-oxours-oleana-9(11),12-dien-28-oic acid (Oxy-Di-OA) was synthesised and investigated for its protective effects against CCl_4_-induced liver injury. The results demonstrated that Oxy-Di-OA could alleviate liver damage. Furthermore, acute toxicity experiments and pharmacokinetic studies revealed that this compound exhibited low toxicity and had a longer half-life when compared with that of OA, indicating its higher bioavailability [40]. Modifications were made to the OA derivative, DKS26, to enable the preparation of lipid nanodiscs (sND/DKS26) and liposomes (sLip/DKS26). Compared to free DKS26, these carrier compounds both exhibited considerably improved oral bioavailability [149]. Luo et al. employed the double emulsion method to prepare multivesicular liposomes encapsulating OA (OA-MVLs). These liposomes were able to increase the solubility of OA and prolong the drug’s residence time in the bloodstream [150]. Simultaneous loading of OA and gentiopicrin into the nanostructured lipid carriers (NLCs) resulted in a stronger effect on liver injury when compared to loading OA alone. Furthermore, the NLCs formulation prolonged the maintenance of drug concentration in the plasma [151]. Therefore the design and synthesis of OA derivatives, utilisation of lipid nano-carriers for OA encapsulation, and combination therapy with other drugs were thus identified as effective approaches to enhance the therapeutic efficacy of OA.

## Summary and outlook

Currently, the available pharmacological treatments for kidney diseases are limited. Two commonly used treatments are ACEI and ARB, which primarily target blood pressure reduction and diuresis to alleviate renal conditions. However, these medications are associated with significant side effects and may exacerbate renal burden. Therefore, identifying new effective and safe drugs is generally considered the best strategy to prevent and treat kidney diseases.

OA demonstrates preventive and therapeutic effects on kidney diseases by modulating various signalling pathways, including NF- κB, STAT, PI3K/AKT, TLR4, and Nrf2/HO-1. Its actions in relation to kidney diseases primarily involve the inhibition of inflammatory responses, immune modulation, attenuation of OS, enhancement of autophagy function, suppression of renal fibrosis, and the reduction of cellular apoptosis. Thus, OA holds promising potential as a therapeutic agent for treating kidney diseases in the future.

Compared with current clinical therapies for kidney diseases, OA offers advantages such as multi-target effects and a high safety profile. However, its poor water solubility, permeability, and low bioavailability may limit its clinical applications. Therefore, further research to design and synthesise OA derivatives and utilise lipid nano-carriers for OA encapsulation is urgently needed to enhance the bioavailability of OA and maximise its therapeutic potential in treating kidney diseases.

## Data Availability

Not applicable.

## Abbreviations

OA: Oleanolic acid
UA: Ursolic acid
NF-κB: Nuclear factor kappa-B
Nrf2: Nuclear factor erythroid2-related factor 2
HO-1: Heme oxygenase-1
TGF-β: Transforming growth factor β
PI3K: Phosphoinositide 3-kinase
AKT/PKB: Protein kinase B
AKI: Acute kidney injury
CKD: Chronic kidney disease
GFR: Glomerular filtration rate
ACE: Angiotensin-converting enzyme inhibitors
ARB: Angiotensin receptor blockers
PRISMA: Preferred Reporting Items for Systematic Reviews and Meta-Analyses
PGI2: Prostacyclin
STZ: Streptozotocin
I/R: Ischemia/reperfusion
UUO: Unilateral ureteral obstruction
CL: Clearance
VSS: Steady-state volume of distribution
AUC: Area under the concentration–time curve
t1/2: Half-life
Cmax: Maximum concentrations
NADPH: Nicotinamide adenine dinucleotide phosphate
Tmax: Time to reach maximum concentration
NO: Nitric oxide
INF-γ: Interferon-γ
CDDO: 2-Cyano-3,12-dioxooleana-1,9-dien-28-oic acid
LC50: Median lethal concentration
LD50: Median lethal dose
IL-1β: Interleukin-1β
IL-6: Interleukin-6
TNF-a: Tumor necrosis factor-α
ICAM: Intercellular cell adhesion molecule
GCLc: Glutamate-cysteine ligase
MDA: Malondialdehyde
SOD: Superoxide dismutase
CAT: Catalase
GSH: Glutathione
LPS: Lipopolysaccharides
TLR4: Toll-like receptor 4
MyD88: Myeloiddifferentiationfactor88
Scr: Serum creatinine
BUN: Blood urea nitrogen
α-SMA: α-Smooth muscle actin
STAT: Signal transducer and activator of transcription
CCl_4_: Carbon tetrachloride
IL-17: Interleukin-17
DN: Diabetic nephropathy
IL-18: Interleukin-18
HG: High glucose
CoL: Collagen
COM: Calcium oxalate monohydrate crystals
LN: Lupus nephritis
IL-2: Interleukin-2
IL-10: Interleukin-10
OS: Oxidative stress
ROS: Reactive oxygen species
RNS: Reactive nitrogen species
8-OHdG: 8-Hydroxy-2′-deoxyguanosine
SOD: Superoxide dismutase
Keap1: Kelch-like ECH-associated protein 1
TβR: TGF-β receptor
EMT: Epithelial-to-mesenchymal transition
PTEN: Phosphatase and tensin homolog
mTOR: Mammalian target of rapamycin
Bcl-2: B-cell lymphoma 2
Bax: BCL2-associated X protein
AGEs: Advanced glycation end products
MAP: Mean arterial pressure
NLCs: Nanostructured lipid carriers

## References

[1] Ostermann M, Bellomo R, Burdmann EA, Doi K, Endre ZH, Goldstein SL (2020). Controversies in acute kidney injury: conclusions from a kidney disease: improving global outcomes (KDIGO) conference. Kidney Int.

[2] Susantitaphong P, Cruz DN, Cerda J, Abulfaraj M, Alqahtani F, Koulouridis I (2013). World incidence of AKI. Clin J Am Soc Nephrol.

[3] Levey AS, de Jong PE, Coresh J, El Nahas M, Astor BC, Matsushita K (2011). The definition, classification, and prognosis of chronic kidney disease: a KDIGO controversies conference report. Kidney Int.

[4] Matsushita K, Ballew SH, Wang AY-M, Kalyesubula R, Schaeffner E, Agarwal R (2022). Epidemiology and risk of cardiovascular disease in populations with chronic kidney disease. Nat Rev Nephrol.

[5] Alicic RZ, Rooney MT, Tuttle KR (2017). Diabetic kidney disease: challenges, progress, and possibilities. Clin J Am Soc Nephrol.

[6] Ku E, Lee BJ, Wei J, Weir MR (2019). Hypertension in CKD: core curriculum 2019. Am J Kidney Dis.

[7] Kovesdy CP, Furth SL, Zoccali C (2017). Obesity and kidney disease: hidden consequences of the epidemic. Blood Purif.

[8] Liu W, Guo R, Huang D, Ji J, Gansevoort RT, Snieder H (2023). Co-occurrence of chronic kidney disease and glaucoma: epidemiology and etiological mechanisms. Surv Ophthalmol.

[9] Jhee JH, Joo YS, Kee YK, Jung S-Y, Park S, Yoon C-Y (2019). Secondhand smoke and CKD. Clin J Am Soc Nephrol.

[10] Winston JA (2010). HIV and CKD epidemiology. Adv Chronic Kidney Dis.

[11] Basile JN (2007). Recognizing the link between CKD and CVD in the primary care setting: accurate and early diagnosis for timely and appropriate intervention. South Med J.

[12] Liyanage T, Toyama T, Hockham C, Ninomiya T, Perkovic V, Woodward M (2022). Prevalence of chronic kidney disease in Asia: a systematic review and analysis. BMJ Glob Health.

[13] Xie Y, Bowe B, Mokdad AH, Xian H, Yan Y, Li T (2018). Analysis of the global burden of disease study highlights the global, regional, and national trends of chronic kidney disease epidemiology from 1990 to 2016. Kidney Int.

[14] Kassebaum NJ, Arora M, Barber RM, Bhutta ZA, Brown J, Carter A (2016). Global, regional, and national disability-adjusted life-years (DALYs) for 315 diseases and injuries and healthy life expectancy (HALE), 1990–2015: a systematic analysis for the global burden of disease study 2015. Lancet.

[15] Rovin BH, Adler SG, Barratt J, Bridoux F, Burdge KA, Chan TM (2021). Executive summary of the KDIGO 2021 guideline for the management of glomerular diseases. Kidney Int.

[16] Khalaf K, Taegtmeyer H (2012). Weight loss surgery, left ventricular mass and repolarization. Am J Cardiol.

[17] Drew DA, Weiner DE, Sarnak MJ (2019). Cognitive impairment in CKD: pathophysiology, management, and prevention. Am J Kidney Dis.

[18] Long C, Yang J, Yang H, Li X, Wang G (2016). Attenuation of renal ischemia/reperfusion injury by oleanolic acid preconditioning via its antioxidant, anti-inflammatory, and anti-apoptotic activities. Mol Med Rep.

[19] Patil CR, Jadhav RB, Singh PK, Mundada S, Patil PR (2010). Protective effect of oleanolic acid on gentamicin induced nephrotoxicity in rats. Phytother Res.

[20] Zhou X, Chen H, Wei F, Zhao Q, Su Q, Lei Y (2020). The inhibitory effects of pentacyclic triterpenes from loquat leaf against Th17 differentiation. Immunol Invest.

[21] Chen J, Cui Y, Zhang N, Yao X, Wang Z, Yang L (2019). Oleanolic acid attenuated diabetic mesangial cell injury by activation of autophagy via miRNA-142-5p/PTEN signaling. Cytotechnology.

[22] Madlala HP, Masola B, Singh M, Musabayane CT (2012). The effects of *Syzygium aromaticum*-derived oleanolic acid on kidney function of male Sprague-Dawley rats and on kidney and liver cell lines. Ren Fail.

[23] Page MJ, McKenzie JE, Bossuyt PM, Boutron I, Hoffmann TC, Mulrow CD (2021). The PRISMA 2020 statement: an updated guideline for reporting systematic reviews. J Clin Epidemiol.

[24] Gudoityte E, Arandarcikaite O, Mazeikiene I, Bendokas V, Liobikas J (2021). Ursolic and oleanolic acids: plant metabolites with neuroprotective potential. Int J Mol Sci.

[25] Liu L, Wang X (2007). Solubility of oleanolic acid in various solvents from (288.3 to 328.3) K. J Chem Eng Data.

[26] Castellano JM, Ramos-Romero S, Perona JS (2022). Oleanolic acid: extraction, characterization and biological activity. Nutrients.

[27] Szakiel A, Pączkowski C, Pensec F, Bertsch C (2012). Fruit cuticular waxes as a source of biologically active triterpenoids. Phytochem Rev.

[28] Allouche Y, Jiménez A, Uceda M, Aguilera MP, Gaforio JJ, Beltrán G (2009). Triterpenic content and chemometric analysis of virgin olive oils from forty olive cultivars. J Agric Food Chem.

[29] Yim TK, Wu WK, Pak WF, Ko KM (2001). Hepatoprotective action of an oleanolic acid-enriched extract of *Ligustrum lucidum* fruits is mediated through an enhancement on hepatic glutathione regeneration capacity in mice. Phytother Res.

[30] Yamaguchi H, Noshita T, Kidachi Y, Umetsu H, Hayashi M, Komiyama K (2008). Isolation of ursolic acid from apple peels and its specific efficacy as a potent antitumor agent. J Health Sci.

[31] Xu X-H, Su Q, Zang Z-H (2012). Simultaneous determination of oleanolic acid and ursolic acid by RP-HPLC in the leaves of *Eriobotrya japonica* Lindl. J Pharm Anal.

[32] Jäger S, Trojan H, Kopp T, Laszczyk M, Scheffler A (2009). Pentacyclic triterpene distribution in various plants—rich sources for a new group of multi-potent plant extracts. Molecules.

[33] Fu Q, Zhang L, Cheng N, Jia M, Zhang YJ (2014). Extraction optimization of oleanolic and ursolic acids from pomegranate (*Punica granatum* L.) flowers. Food Bioprod Process.

[34] Chan TW, But PP, Cheng SW, Kwok IM, Lau FW, Xu HX (2000). Differentiation and authentication of *Panax ginseng*, *Panax quinquefolius*, and ginseng products by using HPLC/MS. Anal Chem.

[35] Fang X, Wang J, Yu X, Zhang G, Zhao J (2010). Optimization of microwave-assisted extraction followed by RP-HPLC for the simultaneous determination of oleanolic acid and ursolic acid in the fruits of *Chaenomeles sinensis*. J Sep Sci.

[36] Banik R, Pandey D (2008). Optimizing conditions for oleanolic acid extraction from *Lantana camara* roots using response surface methodology. Ind Crops Prod.

[37] Domingues RMA, Oliveira ELG, Freire CSR, Couto RM, Simões PC, Neto CP (2012). Supercritical fluid extraction of *Eucalyptus globulus* bark-A promising approach for triterpenoid production. Int J Mol Sci.

[38] Hao B-B, Pan X-X, Fan Y, Lu L, Qian X-F, Wang X-H (2016). Oleanolic acid attenuates liver ischemia reperfusion injury by HO-1/Sesn2 signaling pathway. Hepatobiliary Pancreat Dis Int.

[39] Yan S-l, Huang C-y, Wu S-t, Yin M-c (2010). Oleanolic acid and ursolic acid induce apoptosis in four human liver cancer cell lines. Toxicol In Vitro.

[40] Xiang H, Han Y, Zhang Y, Yan W, Xu B, Chu F (2017). A new oleanolic acid derivative against CCl_4_-induced hepatic fibrosis in rats. Int J Mol Sci.

[41] Liu J (1995). Pharmacology of oleanolic acid and ursolic acid. J Ethnopharmacol.

[42] Bachhav SS, Patil SD, Bhutada MS, Surana SJ (2011). Oleanolic acid prevents glucocorticoid-induced hypertension in rats. Phytother Res.

[43] Martínez-González J, Rodríguez-Rodríguez R, González-Díez M, Rodríguez C, Herrera MD, Ruiz-Gutierrez V (2008). Oleanolic acid induces prostacyclin release in human vascular smooth muscle cells through a cyclooxygenase-2-dependent mechanism. J Nutr.

[44] Feske SK (2021). Ischemic stroke. Am J Med.

[45] Sapkota A, Choi JW (2022). Oleanolic acid provides neuroprotection against ischemic stroke through the inhibition of microglial activation and NLRP3 inflammasome activation. Biomol Ther.

[46] Iskender H, Dokumacioglu E, Terim Kapakin KA, Yenice G, Mohtare B, Bolat I (2022). Effects of oleanolic acid on inflammation and metabolism in diabetic rats. Biotech Histochem.

[47] Rodríguez JA, Astudillo L, Schmeda-Hirschmann G (2003). Oleanolic acid promotes healing of acetic acid-induced chronic gastric lesions in rats. Pharmacol Res.

[48] Peng H-B, Wang R-X, Deng H-J, Wang Y-H, Tang J-D, Cao F-Y (2017). Protective effects of oleanolic acid on oxidative stress and the expression of cytokines and collagen by the AKT/NF-κB pathway in silicotic rats. Mol Med Rep.

[49] Bayülgen A, Ayan E, Gümüş LT, Bozdoğan Arpacı R, Köksel MO (2019). Effect of oleanolic acid for prevention of acute lung injury and apoptosis. Turk Gogus Kalp Damar Cerrahisi Derg.

[50] Kang G-D, Lim S, Kim D-H (2015). Oleanolic acid ameliorates dextran sodium sulfate-induced colitis in mice by restoring the balance of Th17/Treg cells and inhibiting NF-κB signaling pathway. Int Immunopharmacol.

[51] Chung S, Yoon HE, Kim SJ, Kim SJ, Koh ES, Hong YA (2014). Oleanolic acid attenuates renal fibrosis in mice with unilateral ureteral obstruction via facilitating nuclear translocation of Nrf2. Nutr Metab.

[52] Chakravarti B, Maurya R, Siddiqui JA, Bid HK, Rajendran SM, Yadav PP (2012). In vitro anti-breast cancer activity of ethanolic extract of *Wrightia tomentosa*: role of pro-apoptotic effects of oleanolic acid and urosolic acid. J Ethnopharmacol.

[53] Zhu B, Ren C, Du K, Zhu H, Ai Y, Kang F (2019). Olean-28,13b-olide 2 plays a role in cisplatin-mediated apoptosis and reverses cisplatin resistance in human lung cancer through multiple signaling pathways. Biochem Pharmacol.

[54] Zhang X, Wang H, Xu Y, Luan M, Zhao F, Meng Q (2021). Advances on the anti-inflammatory activity of oleanolic acid and derivatives. Mini Rev Med Chem.

[55] Han Y, Wang C, Li X, Liang G (2022). Oleanolic acid reduces oxidative stress and neuronal apoptosis after experimental subarachnoid hemorrhage by regulating Nrf2/HO-1 pathway. Drug Dev Res.

[56] Gutierrez B, Gallardo I, Ruiz L, Alvarez Y, Cachofeiro V, Margolles A (2020). Oleanolic acid ameliorates intestinal alterations associated with EAE. J Neuroinflamm.

[57] Szakiel A, Ruszkowski D, Grudniak A, Kurek A, Wolska KI, Doligalska M (2008). Antibacterial and antiparasitic activity of oleanolic acid and its glycosides isolated from marigold (*Calendula officinalis*). Planta Med.

[58] Kong L, Li S, Liao Q, Zhang Y, Sun R, Zhu X (2013). Oleanolic acid and ursolic acid: novel hepatitis C virus antivirals that inhibit NS5B activity. Antivir Res.

[59] van den Anker J, Reed MD, Allegaert K, Kearns GL (2018). Developmental changes in pharmacokinetics and pharmacodynamics. J Clin Pharmacol.

[60] Jeong DW, Kim YH, Kim HH, Ji HY, Yoo SD, Choi WR (2007). Dose-linear pharmacokinetics of oleanolic acid after intravenous and oral administration in rats. Biopharm Drug Dispos.

[61] Yin M-C, Lin M-C, Mong M-C, Lin C-Y (2012). Bioavailability, distribution, and antioxidative effects of selected triterpenes in mice. J Agric Food Chem.

[62] Lu Y-F, Wan X-L, Xu Y, Liu J (2013). Repeated oral administration of oleanolic acid produces cholestatic liver injury in mice. Molecules.

[63] Pozo OJ, Pujadas M, Gleeson SB, Mesa-García MD, Pastor A, Kotronoulas A (2017). Liquid chromatography tandem mass spectrometric determination of triterpenes in human fluids: evaluation of markers of dietary intake of olive oil and metabolic disposition of oleanolic acid and maslinic acid in humans. Anal Chim Acta.

[64] Li Y, Liu H, Guo B, Li Y, Geng Y, Zhao F (2014). Enhancement of dissolution rate and oral bioavailability in beagle dogs of oleanolic acid by adsorbing onto porous silica using supercritical carbon dioxide. J Drug Deliv Sci Technol.

[65] Saini V, Debnath SK, Maske P, Dighe V, Srivastava R (2022). Targeted delivery of ursolic acid and oleanolic acid to lungs in the form of an inhaler for the management of tuberculosis: pharmacokinetic and toxicity assessment. PLoS ONE.

[66] Ball MS, Bhandari R, Torres GM, Martyanov V, ElTanbouly MA, Archambault K (2020). CDDO-Me alters the tumor microenvironment in estrogen receptor negative breast cancer. Sci Rep.

[67] Hou X, Liu H, Ping Y, Zhang F, Zhi L, Jiang X (2021). CDDO-Im exerts antidepressant-like effects via the Nrf2/ARE pathway in a rat model of post-stroke depression. Brain Res Bull.

[68] Chang PF-M, Acevedo D, Mandarino LJ, Reyna SM (2023). Triterpenoid CDDO-EA inhibits lipopolysaccharide-induced inflammatory responses in skeletal muscle cells through suppression of NF-κB. Exp Biol Med.

[69] Honda T, Finlay HJ, Gribble GW, Suh N, Sporn MB (1997). New enone derivatives of oleanolic acid and ursolic acid as inhibitors of nitric oxide production in mouse macrophages. Bioorgan Med Chem Lett.

[70] Honda T, Rounds BV, Gribble GW, Suh N, Wang Y, Sporn MB (1998). Design and synthesis of 2-cyano-3,12-dioxoolean-1,9-dien-28-oic acid, a novel and highly active inhibitor of nitric oxide production in mouse macrophages. Bioorg Med Chem Lett.

[71] Wang Y-Y, Yang Y-X, Zhe H, He Z-X, Zhou S-F (2014). Bardoxolone methyl (CDDO-Me) as a therapeutic agent: an update on its pharmacokinetic and pharmacodynamic properties. Drug Des Dev Ther.

[72] Honda T, Honda Y, Favaloro FG, Gribble GW, Suh N, Place AE (2002). A novel dicyanotriterpenoid, 2-cyano-3,12-dioxooleana-1,9(11)-dien-28-onitrile, active at picomolar concentrations for inhibition of nitric oxide production. Bioorg Med Chem Lett.

[73] Getachew Y, Cusimano FA, Gopal P, Reisman SA, Shay JW (2016). The synthetic triterpenoid RTA 405 (CDDO-EA) halts progression of liver fibrosis and reduces hepatocellular carcinoma size resulting in increased survival in an experimental model of chronic liver injury. Toxicol Sci.

[74] Liby K, Royce DB, Williams CR, Risingsong R, Yore MM, Honda T (2007). The synthetic triterpenoids CDDO-methyl ester and CDDO-ethyl amide prevent lung cancer induced by vinyl carbamate in A/J mice. Can Res.

[75] So JY, Lin JJ, Wahler J, Liby KT, Sporn MB, Suh N (2014). A synthetic triterpenoid CDDO-Im inhibits tumorsphere formation by regulating stem cell signaling pathways in triple-negative breast cancer. PLoS ONE.

[76] Kim E-H, Deng C-X, Sporn MB, Liby KT (2011). CDDO-imidazolide induces DNA damage, G2/M arrest and apoptosis in BRCA1-mutated breast cancer cells. Cancer Prev Res.

[77] Kocak C, Kocak FE, Akcilar R, Bayat Z, Aras B, Metineren MH (2016). Effects of captopril, telmisartan and bardoxolone methyl (CDDO-Me) in ischemia-reperfusion-induced acute kidney injury in rats: an experimental comparative study. Clin Exp Pharmacol Physiol.

[78] Liu M, Reddy NM, Higbee EM, Potteti HR, Noel S, Racusen L (2014). The Nrf2 triterpenoid activator, CDDO-imidazolide, protects kidneys from ischemia-reperfusion injury in mice. Kidney Int.

[79] Song M-K, Lee J-H, Ryoo I-G, Lee S-H, Ku S-K, Kwak M-K (2019). Bardoxolone ameliorates TGF-β1-associated renal fibrosis through Nrf2/Smad7 elevation. Free Radic Biol Med.

[80] Somova L, Nadar A, Rammanan P, Shode FO (2003). Cardiovascular, antihyperlipidemic and antioxidant effects of oleanolic and ursolic acids in experimental hypertension. Phytomedicine.

[81] Garrido JC, Cevallos GAC, Siciliano LG, Pando RH, Arellanes MA (2012). Acute and subacute toxicity (28 days) of a mixture of ursolic acid and oleanolic acid obtained from *Bouvardia ternifolia* in mice. Boletín Latinoamericano y del Caribe de Plantas Medicinales y aromaticas.

[82] Xu L, Wan Z (1980). The effect of oleanolic acid on acute hepatitis (70 cases). Hum Med.

[83] Turgut F, Awad AS, Abdel-Rahman EM (2023). Acute kidney injury: medical causes and pathogenesis. J Clin Med.

[84] Seeliger E, Sendeski M, Rihal CS, Persson PB (2012). Contrast-induced kidney injury: mechanisms, risk factors, and prevention. Eur Heart J.

[85] Liu C, Yan S, Wang Y, Wang J, Fu X, Song H (2021). Drug-induced hospital-acquired acute kidney injury in China: a multicenter cross-sectional survey. Kidney Dis.

[86] Abdel-Zaher AO, Abdel-Rahman MM, Hafez MM, Omran FM (2007). Role of nitric oxide and reduced glutathione in the protective effects of aminoguanidine, gadolinium chloride and oleanolic acid against acetaminophen-induced hepatic and renal damage. Toxicology.

[87] Aleksunes LM, Goedken MJ, Rockwell CE, Thomale J, Manautou JE, Klaassen CD (2010). Transcriptional regulation of renal cytoprotective genes by Nrf2 and its potential use as a therapeutic target to mitigate cisplatin-induced nephrotoxicity. J Pharmacol Exp Ther.

[88] Zhao J, Zheng H, Sui Z, Jing F, Quan X, Zhao W (2019). Ursolic acid exhibits anti-inflammatory effects through blocking TLR4-MyD88 pathway mediated by autophagy. Cytokine.

[89] Zhou P, Xue C, Li Y, Li C, Hu X, Liu Y (2011). A novel NF-κB inhibitor attenuates renal ischemia–reperfusion injury in mice. Am J Transplant.

[90] Ammirati AL (2020). Chronic kidney disease. Rev Assoc Med Bras.

[91] Drawz P, Rahman M (2015). Chronic kidney disease. Ann Intern Med.

[92] Crews DC, Bello AK, Saadi G (2019). Burden, access, and disparities in kidney disease. Blood Purif.

[93] Pergola PE, Krauth M, Huff JW, Ferguson DA, Ruiz S, Meyer CJ (2011). Effect of bardoxolone methyl on kidney function in patients with T2D and stage 3b–4 CKD. Am J Nephrol.

[94] Wei M, Yin J (2017). Oleanolic acid alleviates renal fibrosis through regulating the expression of MIR-141. J Am Soc Nephrol.

[95] Ma T-K, Xu L, Lu L-X, Cao X, Li X, Li L-L (2019). Ursolic acid treatment alleviates diabetic kidney injury by regulating the ARAP1/AT1R signaling pathway. Diabetes Metab Syndr Obe Targets Ther.

[96] Meng X-M (2019). Inflammatory mediators and renal fibrosis. Adv Exp Med Biol.

[97] Meng X-M, Nikolic-Paterson DJ, Lan HY (2014). Inflammatory processes in renal fibrosis. Nat Rev Nephrol.

[98] Kang Y-M, Lee M, An H-J (2021). Oleanolic acid protects against mast cell-mediated allergic responses by suppressing Akt/NF-κB and STAT1 activation. Phytomed Int J Phytother Phytopharmacol.

[99] Dong N, Xue C, Zhang L, Zhang T, Wang C, Bi C (2020). Oleanolic acid enhances tight junctions and ameliorates inflammation in *Salmonella typhimurium*-induced diarrhea in mice via the TLR4/NF-κB and MAPK pathway. Food Funct.

[100] Ma JQ, Ding J, Xiao ZH, Liu CM (2014). Ursolic acid ameliorates carbon tetrachloride-induced oxidative DNA damage and inflammation in mouse kidney by inhibiting the STAT3 and NF-κB activities. Int Immunopharmacol.

[101] Peng J, Ren X, Lan T, Chen Y, Shao Z, Yang C (2016). Renoprotective effects of ursolic acid on ischemia/reperfusion-induced acute kidney injury through oxidative stress, inflammation and the inhibition of STAT3 and NF-κB activities. Mol Med Rep.

[102] Potočnjak I, Šimić L, Vukelić I, Domitrović R (2019). Oleanolic acid attenuates cisplatin-induced nephrotoxicity in mice and chemosensitizes human cervical cancer cells to cisplatin cytotoxicity. Food Chem Toxicol.

[103] Liu Y, Hu Z, Xing H, Kang L, Chen X, Liu B (2022). Renoprotective effects of oleanolic acid and its possible mechanisms in rats with diabetic kidney disease. Biochem Biophys Res Commun.

[104] Jia Z, Li W, Bian P, Yang L, Liu H, Pan D (2021). Ursolic acid treats renal tubular epithelial cell damage induced by calcium oxalate monohydrate via inhibiting oxidative stress and inflammation. Bioengineered.

[105] Li J, Li N, Yan S, Liu M, Sun B, Lu Y (2018). Ursolic acid alleviates inflammation and against diabetes-induced nephropathy through TLR4-mediated inflammatory pathway. Mol Med Rep.

[106] Kurts C, Panzer U, Anders H-J, Rees AJ (2013). The immune system and kidney disease: basic concepts and clinical implications. Nat Rev Immunol.

[107] Tecklenborg J, Clayton D, Siebert S, Coley SM (2018). The role of the immune system in kidney disease. Clin Exp Immunol.

[108] Lech M, Anders H-J (2013). The pathogenesis of lupus nephritis. J Am Soc Nephrol.

[109] Zhou X, Chen H, Wei F, Zhao Q, Su Q, Liang J (2019). 3β-Acetyloxy-oleanolic acid attenuates pristane-induced lupus nephritis by regulating Th17 differentiation. J Immunol Res.

[110] Nataraju A, Saini D, Ramachandran S, Benshoff N, Liu W, Chapman W (2009). Oleanolic acid, a plant triterpenoid, significantly improves survival and function of islet allograft. Transplantation.

[111] Qian K, Liao W, Li J, Jiang H, Zhou H, Long J (2014). Oleanolic acid synergizes with cyclosporine A to prolong renal allograft survival in rats. Nan fang yi ke da xue xue bao = J South Med Univ.

[112] Ullevig SL, Kim HS, Nguyen HN, Hambright WS, Robles AJ, Tavakoli S (2014). Ursolic acid protects monocytes against metabolic stress-induced priming and dysfunction by preventing the induction of Nox4. Redox Biol.

[113] Halliwell B (2007). Biochemistry of oxidative stress. Biochem Soc Trans.

[114] Su H, Wan C, Song A, Qiu Y, Xiong W, Zhang C (2019). Oxidative stress and renal fibrosis: mechanisms and therapies. Adv Exp Med Biol.

[115] Bhargava P, Schnellmann RG (2017). Mitochondrial energetics in the kidney. Nat Rev Nephrol.

[116] Khan MA, Wang X, Giuliani KTK, Nag P, Grivei A, Ungerer J (2020). Underlying histopathology determines response to oxidative stress in cultured human primary proximal tubular epithelial cells. Int J Mol Sci.

[117] Brealey D, Brand M, Hargreaves I, Heales S, Land J, Smolenski R (2002). Association between mitochondrial dysfunction and severity and outcome of septic shock. Lancet.

[118] Dounousi E, Papavasiliou E, Makedou A, Ioannou K, Katopodis KP, Tselepis A (2006). Oxidative stress is progressively enhanced with advancing stages of CKD. Am J Kidney Dis.

[119] Wang X, Ye X-L, Liu R, Chen H-L, Bai H, Liang X (2010). Antioxidant activities of oleanolic acid in vitro: possible role of Nrf2 and MAP kinases. Chemico-biol Interact.

[120] Thimmulappa RK, Fuchs RJ, Malhotra D, Scollick C, Traore K, Bream JH (2007). Preclinical evaluation of targeting the Nrf2 pathway by triterpenoids (CDDO-Im and CDDO-Me) for protection from LPS-induced inflammatory response and reactive oxygen species in human peripheral blood mononuclear cells and neutrophils. Antioxid Redox Signal.

[121] Gatbonton-Schwager T, Yagishita Y, Joshi T, Wakabayashi N, Srinivasan H, Suzuki T (2023). A point mutation at C151 of Keap1 of mice abrogates NRF2 signaling, cytoprotection in vitro, and hepatoprotection in vivo by bardoxolone methyl (CDDO-Me). Mol Pharmacol.

[122] Pergola PE, Raskin P, Toto RD, Meyer CJ, Huff JW, Grossman EB (2011). Bardoxolone methyl and kidney function in CKD with type 2 diabetes. N Engl J Med.

[123] Rossing P, Block GA, Chin MP, Goldsberry A, Heerspink HJL, McCullough PA (2019). Effect of bardoxolone methyl on the urine albumin-to-creatinine ratio in patients with type 2 diabetes and stage 4 chronic kidney disease. Kidney Int.

[124] Lee ES, Kim HM, Kang JS, Lee EY, Yadav D, Kwon M-H (2016). Oleanolic acid and *N*-acetylcysteine ameliorate diabetic nephropathy through reduction of oxidative stress and endoplasmic reticulum stress in a type 2 diabetic rat model. Nephrol Dial Transplant.

[125] Hong YA, Lim JH, Kim MY, Kim EN, Koh ES, Shin SJ (2014). Delayed treatment with oleanolic acid attenuates tubulointerstitial fibrosis in chronic cyclosporine nephropathy through Nrf2/HO-1 signaling. J Transl Med.

[126] Kurosaki Y, Imoto A, Kawakami F, Ouchi M, Morita A, Yokoba M (2022). In vitro study on effect of bardoxolone methyl on cisplatin-induced cellular senescence in human proximal tubular cells. Mol Cell Biochem.

[127] Nastase MV, Zeng-Brouwers J, Wygrecka M, Schaefer L (2018). Targeting renal fibrosis: mechanisms and drug delivery systems. Adv Drug Deliv Rev.

[128] Humphreys BD (2018). Mechanisms of renal fibrosis. Annu Rev Physiol.

[129] Kim M-S, Han J-Y, Kim S-H, Jeon D, Kim H-Y, Lee SW (2018). Oleanolic acid acetate attenuates polyhexamethylene guanidine phosphate-induced pulmonary inflammation and fibrosis in mice. Respir Physiol Neurobiol.

[130] Martín R, Cordova C, San Román JA, Gutierrez B, Cachofeiro V, Nieto ML (2014). Oleanolic acid modulates the immune-inflammatory response in mice with experimental autoimmune myocarditis and protects from cardiac injury. Therapeutic implications for the human disease. J Mol Cell Cardiol.

[131] Zhao D, Luan Z (2020). Oleanolic acid attenuates renal fibrosis through TGF-β/Smad pathway in a rat model of unilateral ureteral obstruction. Evid-based Complement Altern Med.

[132] Xu C-G, Zhu X-L, Wang W, Zhou X-J (2019). Ursolic acid inhibits epithelial–mesenchymal transition in vitro and in vivo. Pharm Biol.

[133] He W-M, Yin J-Q, Cheng X-D, Lu X, Ni L, Xi Y (2018). Oleanolic acid attenuates TGF-β1-induced epithelial–mesenchymal transition in NRK-52E cells. BMC Complement Altern Med.

[134] Glick D, Barth S, Macleod KF (2010). Autophagy: cellular and molecular mechanisms. J Pathol.

[135] Kimura T, Takabatake Y, Takahashi A, Kaimori J-Y, Matsui I, Namba T (2011). Autophagy protects the proximal tubule from degeneration and acute ischemic injury. J Am Soc Nephrol.

[136] Kim W-Y, Nam SA, Song HC, Ko JS, Park SH, Kim HL (2012). The role of autophagy in unilateral ureteral obstruction rat model. Nephrology.

[137] Lu X, Fan Q, Xu L, Li L, Yue Y, Xu Y (2015). Ursolic acid attenuates diabetic mesangial cell injury through the up-regulation of autophagy via miRNA-21/PTEN/Akt/mTOR suppression. PLoS ONE.

[138] Xu L, Fan Q (2015). Ursolic acid improves the podocytes injury caused by high glucose. Hong Kong J Nephrol.

[139] Elmore S (2007). Apoptosis: a review of programmed cell death. Toxicol Pathol.

[140] Sanz AB, Sanchez-Niño MD, Ramos AM, Ortiz A (2023). Regulated cell death pathways in kidney disease. Nat Rev Nephrol.

[141] Tripathi P, Alshahrani S (2021). Mitigation of ILβ-1, ILβ-6, TNF-α, and markers of apoptosis by ursolic acid against cisplatin-induced oxidative stress and nephrotoxicity in rats. Hum Exp Toxicol.

[142] Zhang Q, Chen W, Zhang B, Zhang Y, Xiao Y, An Y (2023). Lonp1 and Sig-1R contribute to the counteraction of ursolic acid against ochratoxin A-induced mitochondrial apoptosis. Food Chem Toxicol.

[143] Alqrad MAI, El-Agamy DS, Ibrahim SRM, Sirwi A, Abdallah HM, Abdel-Sattar E (2023). SIRT1/Nrf2/NF-κB signaling mediates anti-inflammatory and anti-apoptotic activities of oleanolic acid in a mouse model of acute hepatorenal damage. Medicina.

[144] Inagi R (2021). Organelle stress and glycation in kidney disease. Glycoconj J.

[145] Schjoldager KT, Narimatsu Y, Joshi HJ, Clausen H (2020). Global view of human protein glycosylation pathways and functions. Nat Rev Mol Cell Biol.

[146] Wang ZH, Hsu CC, Huang CN, Yin MC (2010). Anti-glycative effects of oleanolic acid and ursolic acid in kidney of diabetic mice. Eur J Pharmacol.

[147] Mapanga RF, Tufts MA, Shode FO, Musabayane CT (2009). Renal effects of plant-derived oleanolic acid in streptozotocin-induced diabetic rats. Ren Fail.

[148] Madlala HP, Van Heerden FR, Mubagwa K, Musabayane CT (2015). Changes in renal function and oxidative status associated with the hypotensive effects of oleanolic acid and related synthetic derivatives in experimental animals. PLoS ONE.

[149] Ban Y, Chu Y, Pan F, Guo Z, Yang Y, Wei X (2023). Lipid-based nanocarriers enabled oral delivery of oleanolic acid derivative DKS26 for diabetes management. Adv Healthc Mater.

[150] Luo Y, Liu Z, Zhang X, Huang J, Yu X, Li J (2016). Effect of a controlled-release drug delivery system made of oleanolic acid formulated into multivesicular liposomes on hepatocellular carcinoma in vitro and in vivo. Int J Nanomed.

[151] Zhang K, Lv S, Li X, Feng Y, Li X, Liu L (2013). Preparation, characterization, and in vivo pharmacokinetics of nanostructured lipid carriers loaded with oleanolic acid and gentiopicrin. Int J Nanomed.

[152] Song M, Hang T-J, Wang Y, Jiang L, Wu X-L, Zhang Z (2006). Determination of oleanolic acid in human plasma and study of its pharmacokinetics in Chinese healthy male volunteers by HPLC tandem mass spectrometry. J Pharm Biomed Anal.

[153] Rada M, Castellano JM, Perona JS, Guinda Á (2015). GC-FID determination and pharmacokinetic studies of oleanolic acid in human serum. Biomed Chromatogr.

[154] Zhou Y, Li JS, Zhang X, Wu YJ, Huang K, Zheng L (2010). Ursolic acid inhibits early lesions of diabetic nephropathy. Int J Mol Med.

[155] Wu QQ, Wang Y, Senitko M, Meyer C, Wigley WC, Ferguson DA (2011). Bardoxolone methyl (BARD) ameliorates ischemic AKI and increases expression of protective genes Nrf2, PPARγ, and HO-1. Am J Physiol Renal Physiol.

[156] Angaswamy N, Tiriveedhi V, Banan B, Benshoff N, Chapman W, Mohanakumar T (2013). Synergism of a natural plant product, oleanolic acid with calcineurin inhibitor in prolonging islet allograft survival. Transpl Immunol.

[157] Dubey VK, Patil CR, Kamble SM, Tidke PS, Patil KR, Maniya PJ (2013). Oleanolic acid prevents progression of streptozotocin induced diabetic nephropathy and protects renal microstructures in Sprague Dawley rats. J Pharmacol Pharmacother.

[158] Ling C, Jinping L, Xia L, Renyong Y (2013). Ursolic acid provides kidney protection in diabetic rats. Curr Ther Res Clin Exp.

[159] Wu J, Liu X, Fan J, Chen W, Wang J, Zeng Y (2014). Bardoxolone methyl (BARD) ameliorates aristolochic acid (AA)-induced acute kidney injury through Nrf2 pathway. Toxicology.

[160] Ding Y-J, Sun C-Y, Wen C-C, Chen Y-H (2015). Nephroprotective role of resveratrol and ursolic acid in aristolochic acid intoxicated zebrafish. TOXINS.

[161] Bacanli M, Aydin S, Anlar HG, Cal T, Bucurgat UU, Ari N (2018). Protective effects of ursolic acid in the kidneys o diabetic rats. Turk J Pharm Sci.

[162] Thakur R, Sharma A, Lingaraju MC, Begum J, Kumar D, Mathesh K (2018). Ameliorative effect of ursolic acid on renal fibrosis in adenine-induced chronic kidney disease in rats. Biomed Pharmacother.

[163] Xu H-L, Wang X-T, Cheng Y, Zhao J-G, Zhou Y-J, Yang J-J (2018). Ursolic acid improves diabetic nephropathy via suppression of oxidative stress and inflammation in streptozotocin-induced rats. Biomed Pharmacother.

[164] Zhang Z, Zhang H, Chen R, Wang Z (2018). Oral supplementation with ursolic acid ameliorates sepsis-induced acute kidney injury in a mouse model by inhibiting oxidative stress and inflammatory responses. Mol Med Rep.

[165] Li C, Chen W, Zheng L, Zhang B, Yang X, Zhang Q (2019). Ameliorative effect of ursolic acid on ochratoxin A-induced renal cytotoxicity mediated by Lonp1/Aco2/Hsp75. Toxicon.

[166] Zheng J, Zhang S, Chen H, Cai X, Zhang C, Li S (2020). Protosappanin-A and oleanolic acid protect injured podocytes from apoptosis through inhibition of AKT-mTOR signaling. Cell Biol Int.

[167] Wu X, Li H, Wan Z, Wang R, Liu J, Liu Q (2021). The combination of ursolic acid and empagliflozin relieves diabetic nephropathy by reducing inflammation, oxidative stress and renal fibrosis. Biomed Pharmacother.

[168] Yang J, Li X, Yang H, Long C (2021). Oleanolic acid improves the symptom of renal ischemia reperfusion injury via the PI3K/AKT pathway. Urol Int.

[169] Zhang Q, Chen W, Zhang B, Li C, Zhang X, Wang Q (2021). Central role of TRAP1 in the ameliorative effect of oleanolic acid on the mitochondrial-mediated and endoplasmic reticulum stress-excitated apoptosis induced by ochratoxin A. Toxicology.

[170] Liu Y, Zheng JY, Wei ZT, Liu SK, Sun JL, Mao YH (2022). Therapeutic effect and mechanism of combination therapy with ursolic acid and insulin on diabetic nephropathy in a type I diabetic rat model. Front Pharmacol.

[171] Pei J, Wu M, Cai S, Peng J, Zhan X, Wang D (2022). The protective effect of ursolic acid on unilateral ureteral obstruction in rats by activating the Nrf2/HO-1 antioxidant signaling pathway. Comput Intell Neurosci.

